# ISSPP CONGRESS 2022 3RD CONGRESS OF THE INTERNATIONAL SOCIETY FOR THE STUDY OF PLEURA AND PERITONEUM

**DOI:** 10.1515/pp-2023-0010

**Published:** 2023-03-29

Huntington Beach, CA, U.S.A.

October 13-15, 2022

**Co-Chairs of the Organizing Committee**

Thanh Dellinger, M.D., Associate Professor, Division of Gynecologic Oncology

Mustafa Raoof, M.D., Assistant Professor, Division of Surgical Oncology

Yanghee Woo, M.D., Associate Professor, Division of Surgical Oncology (Guest Editor)

Department of Surgery, City of Hope National Medical Center, Duarte, CA, U.S.A

Co-Hosted by Dr. Yuman Fong

Sangiacomo Chair, Department of Surgery

City of Hope National Medical Center, Duarte Ca, U.S.A

&

Dr. Robert Ramsay, President of ISSPP

**PLENARY ASBTACTS**

## ISSSPP 2022 PLENARY ABSTRACT 1.

### PIPAC ESTOK 01: FIRST RANDOMIZED AND MULTICENTER PHASE II STUDY ON DOXORUBICIN/CISPLATIN PRESSURIZED INTRA PERITONEAL AEROSOL CHEMOTHERAPY IN GASTRIC PERITONEAL METASTASIS: FIRST RESULTS ON EARLY POSTOPERATIVE OUTCOMES

Eveno, Clarisse Eveno^1^, Cécile Br^i^gand^2^, Olivia Sgarbura^3^, Marc Pocard^4^

^1^Lille University Hospital Center, Kepenekian, Vahan; Centre Hospitalier Lyon-Sud, Surgical Oncology; ^2^Hautepierre Hospital, Strasbourg University Hospital, Department of General and Digestive Surgery De Franco, Valeria; ^3^Regional Cancer Research Institute Montpellier, Surgical Oncology Durand Fontanier, Sylvaine; ^4^Paris 7 University, Surgical unit; INSERM, U965 CART unit

**Introduction**: PIPAC is a novel intraperitoneal drug delivery method that has shown promising results in larges retrospectives studies. Our aim was to analyze the effect on safety and survival of the adjunction of PIPAC to intravenous chemotherapy.

**Methods**: PIPAC EstoK 01 is a prospective, open, randomized multicenter phase II clinical study with two arms that aims at evaluating the effects of PIPAC with doxorubicin and cisplatin (D/C) on patients with PM of gastric cancer with peritoneal cancer index (PCI) > 8. Chemo arm receive 9 cycles of Chemo. Arm PIPAC+Chemo received 3 PIPACs incorporated with 6 Chemo (2 cycles between two PIPAC). Primary endpoint was progression-free survival from the date of randomization to the date of death. Secondary endpoint was 2-year overall survival, morbidity, quality of life and secondary resectability rate. The number of patients randomized was calculated to be 94.

**Results**: 31 in patients in chemo arm and 33 in PIPAC+chemo arm were enrolled. 104 PIPAC applications were performed with 18 (58%) of patients that completed 3 cycles. Mean age were 56.5 [49-65], PCI 23 [15-28], with 26% of patients that had previous surgery and 14% of patients having received at least 2 lines of prior chemotherapy and 54% having ascites during first laparoscopy. Major morbidity and postoperative mortality rate were 22.5 and 3.3% in chemo arm and 18.2 and 0% PIPAC+chemo arm; bowel obstruction underwent in 35.5 and 45.5% respectively and was fund to be due to progressive disease in 91 end 93% respectively and responsible for the patient’s death in 36.4 and 73.3% respectively. Patient’s enrollment was stopped by authorities for PIPAC toxicity regarding a possible increased risk of death by occlusion in the PIPAC+Chemo arm.

**Conclusions**: Our study is the first randomized trial testing D/C PIPAC on gastric carcinomatosis in palliative settings, associated with Chemo IV. Better selection of patients should be performed to allow patients to complete 3 cycles of PIPAC and avoid bowel obstruction with rapid progressive disease. Long term results are awaited.

## ISSPP 2022 PLENARY ABSTRACT 2.

### COMBINATION OF OXALIPLATIN+ATRI+ANTI-PD1 FOR THE TREATMENT OF PERITONEAL METASTASIS OF COLORECTAL ORIGIN IS HIGHLY EFFECTIVE

Alexandra Fauvre^1^, Laura Jeanson^1^, Julie Constanzo^1^, Salima Atis^1^, Marion Larroque^1^, Nadia Vie^1^, Lakhdar Khellaf^2^, Diego Tosi1,^3^, Olivia Sgarbura^1,4^, Céline Gongora^1^

^1^Institut du Recherche dans le Cancer Montpellier (IRCM) INSERM U1194, Faculté de Médecine, Montpellier France; ^2^ Institut du Cancer Montpellier (ICM) Pathology Department, Faculté de Médecine, Montpellier France; ^3^ Institut du Cancer Montpellier (ICM) Medical Oncology Department, Faculté de Médecine, Montpellier France; ^4^ Institut du Cancer Montpellier (ICM) Surgical Oncology Department, Faculté de Médecine, Montpellier France

**Introduction**: Peritoneal metastases (PM) are a common site for colorectal cancer (CRC). While Prodige7 showed few benefits of oxaliplatin-based HIPEC, one of the potential explanations is acquired resistance to oxaliplatin. We recently identified the ATR kinase as a new target involved in oxaliplatin resistance. We demonstrated that the combination of ATR inhibitor with oxaliplatin (VOX treatment) exhibited a synergistic antitumor effect. Our hypothesis was then to combine VOX treatment with immunotherapy in a mouse model of PM.

**Methods**: We used two models of PM: MC-38 colorectal cancer cells grafted into C57BL/6 mice and CT-26 into Balb/c mice. We first looked for the expression of PD-L1 on the surface of MC38 and CT26 cells before and after treatment with VOX. For the in-vivo experiments, we grafted respectively 50,000 MC38 cells into C57BL/6 and 100,000 CT-26 into Balb/c mice. The two cell lines expressed constitutively the luciferase gene to detect the tumor mass using chemiluminescence. 6 treatment groups for each model: NT, Oxaliplatin 5 mg/kg once a week IP, VX-970 30 mg/kg twice a week IP, Oxaliplatin + VX-970, anti-PD1 200 µg once per week IP and Oxaliplatin+VX-970+anti-PD1. Tumor growth has been assessed once a week by chemiluminescence.

**Results**: We observed that PD-L1 is expressed in MC38 and CT26, and that its expression is increased in vitro after treatment with oxaliplatin or VOX. This indicates that a combination of VOX with an anti-PD1 is feasible and may have a synergistic effect. Then we tested the combination in the two in-vivo models MC38 and CT26. Oxaliplatin and VOX led to complete regression of the tumors in 44% of mice while anti-PD1 and VOX+anti-PD1 lead to complete regression of the tumors in 66% of mice. For the CT26, we observed 100% of mice with undetectable tumors when treated with VOX+anti-PD1. Re-challenge is ongoing.

**Conclusions**: Our experiments showed that the combination of oxaliplatin+ATR inhibitor (VOX)+ anti-PD1 is highly efficient to treat PM and induces a long-term anti-tumor immunity in two different in vivo models. These results suggest that this treatment can be tested in a clinical trial.

## ISSPP 2022 PLENARY ABSTRACT 3.

### COMBINATION OF ANTIANGIOGENIC ANTIBIOTIC TREATMENT INHIBITS THE GROWTH OF PSEUDOMYXOMA PERITONEI IN VIVO IN A MOUSE MODEL

Cynthia Crocheray^1^ and Marc Pocard^2^

^1^Université de Paris Cité, UMR U1275 CAP Paris Techno - Lariboisière Hospital; ^2^Paris 7 University, Surgical unit ; INSERM, U965 CART unit

**Introduction**: Pseudomyxoma peritonei (PMP), is a rare cancer of the peritoneum. Primary tumor, as it develops in the lumen of the appendix, would clog the appendix and lead to an accumulation of mucus, which would lead to the rupture of the appendix in the peritoneum cavity. This origin then raises the hypothesis of a bacterial component associated with tumor cells. In addition, many vessels are found in this disease. These two components have led our unit to explore the effect of antibiotics and anti-angiogenic combination drugs on the development of PMP.

**Methods**: To obtain a mouse model of PMP, we performed an intraperitoneal transplant to collect patients in the abdomen of immunosuppressive nude mice (Apafis project #6771 authorized by the Ministry of Higher Education and Research). Two models were obtained: model STLOM with solid PMP and model BOGH in the form of gelatinous ascites. We tested Aflibercept (25mg/kg 2 times a week) - Penicillin/Streptavidin (20mg/kg 5 times a week) combination drugs. Treatment began 6 days and 7 days respectively post-transplant and the experiment finished 41 days and 45 days respectively after transplantation. The blood and tumor (and ascites) were recovered at the end of the experiment for biochemical analyses of our post-mortem project. VEGF was assayed by ELISA and ACE by electrochemistoluminescence (ECLIA).

**Results**: We were able to observe for both PMP models a significant difference between mice treated with Aflibercept and Penicillin/Streptomycin vs control mice. Indeed, the median weight of the tumor STLOM (0,767g for treated mice vs 3,364g for control mice) or the median volume of ascites BOGH (0,0445ml for treated mice vs 3,867ml for control mice) obtained is significantly lower for the treated mice (p<0.0001). In addition, for the latter, the median concentration of ACE is 4 times lower (12,33ng/ml for treated mice vs 51,36ng/ml for control mice in STLOM model (p=0,0155) and 7,8ng/ml for treated mice vs 30,15ng/ml for control mice in BOGH model (p=0,0012)). Finally, median serum and tumor VEGF concentrations per microgram of protein are significantly higher in mice treated with the combination (7,909pg/ml in the serum and 39,55pg/ml in the tumor of STLOM model; 9,273pg/ml in the serum and 28,06pg/ml in the ascites of BOGH model) compared to control mice (0,1387pg/ml in the serum (p<0,0001) and 6,177pg/ml in the tumor (p=0,0003) of STLOM model; 0,1682pg/ml in the serum (p<0,0001) and 0,3498pg/ml in the ascites (p=0,0001) of BOGH model).

**Conclusions**: The anti-angiogenic antibiotic combination appears to be effective in inhibiting the growth of PMP *in vivo* in mice models. It could be used as non-chemotherapy treatment for patients who could not withstand cytoreductive surgery.

## ISSPP 2022 PLENARY ABSTRACT 4.

### A SINGLE-CELL RESOLUTION LANDSCAPE OF PERITONEAL METASTASES OF COLORECTAL CANCER

Jesse Demuytere^1,3^, Jef Haerinck^2,3^, Jordy De Coninck^2,3^, Sam Ernst^1,3^, Joachim Taminau^2,3^, Wim Ceelen^1,3^, Geert Berx^2,3^

^1^Laboratory of experimental surgery, Department of human structure and repair, Ghent University, Ghent, Belgium; ^2^Molecular and Cellular Oncology Laboratory, Department of Biomedical Molecular Biology, Ghent University, Ghent, Belgium; ^3^Cancer Research Institute Ghent (CRIG), Ghent, Belgium

**Introduction**: Peritoneal metastases (PM) of colorectal cancer (CRC) are predominantly described as exhibiting a CMS4 subtype, which is characterized by mesenchymal activation and an abundant tumour microenvironment (TME). Single cell transcriptomics allow for mapping the microenvironment with high fidelity, allowing novel insights into the role of the TME in peritoneal metastasis.

**Methods**: We prospectively gathered fresh tissue samples from 8 HIPEC-naïve patients with CRC PM at four distinct anatomic locations when possible (primary tumor, abdominal wall, bowel mesentery and omentum). After tissue dissociation using a combined enzymatic and mechanical method and FACS, we performed library prep and single cell RNA sequencing on the Chromium platform (10x Genomics). After necessary quality control and clustering with the Cellranger pipeline, we performed detailed annotation of identified cell clusters.

**Results**: We analyzed 23 samples, sequencing 126316 cells. Generally, samples demonstrated large CAF and immune cell clusters, with distinct functional subclusters. We confirmed the presence of subtypes of CAFs, namely an extracellular matrix remodelling subtype (characterised by upregulation of collagen, proteoglycans and matrix metalloproteases) and a vascular CAF subtype associated with wound healing and neoangiogenesis. In addition to CAFs, large macrophage clusters were present, with both classical monocyte clusters and CAM subclusters readily defined. Large T-cell populations indicated a “hot” immune microenvironment, with Th and cytotoxic T-cells present. In comparison to primary tumour samples, tumour-infiltrating B cells were rarely observed in matched metastatic tissue, whereas large plasma cell clusters were apparent in our matched primary tissues. Furthermore, primary tumour samples demonstrated large numbers of epithelial cells in comparison to PM. Both inter- and intrapatient heterogeneity was high, demonstrating the need for targeted anatomical sampling.

**Conclusion**: Single-cell RNA sequencing demonstrates a predominantly mesenchymal microenvironment of CRC PM. Further analysis of our dataset will allow novel insights into the dynamics and unique features of peritoneal metastasis and its TME.

## ISSPP 2022 PLENARY ABSTRACT 5.

### INTRAOPERATIVE PHOTODYNAMIC DIAGNOSTIC WITH HYPERICIN IN GASTRIC CANCER – A PILOT TRIAL

Can Yurttas^1^, Marcus W Löffler^1-5^, Karolin Thiel K^1^, Ruth Ladurner^1^, Ingmar Königsrainer^1^, ^7^, Alfred Königsrainer^1-3^, Philip Horvath^1^, Stefan Beckert^1-6^

^1^Department of General, Visceral and Transplant Surgery, University Hospital Tübingen, Germany; ^2^German Cancer Consortium (DKTK) and German Cancer Research Center (DKFZ) Partner Site Tübingen, Tübingen, Germany; ^3^Cluster of Excellence iFIT (EXC2180) "Image-Guided and Functionally Instructed Tumor Therapies", University of Tübingen, Tübingen, Germany; ^4^Interfaculty Institute for Cell Biology, Department of Immunology, University of Tübingen, Turbingen, Germany; ^5^Department of Clinical Pharmacology, University Hospital Tübingen, Tübingen, Germany; ^6^Department of General and Visceral Surgery, Villingen-Schwenningen, Germany; ^7^Department of General, Visceral and Thoracic Surgery, Feldkirch, Austria

**Introduction**: Laparoscopy is an essential element of staging in locally advanced gastric cancer indispensable for therapy planning. However, differentiation of tumor from scar tissue often requires biopsy. Hypericin, which is contained in St. John´s Wort, shows fluorescence when excited by ultraviolet light as well as cytotoxic properties. Since tumor cells accumulate hypericin, utilization of photodynamic diagnostics to guide biopsy in gastric cancer has been suggested.

**Methods**: Patients scheduled for staging laparoscopy with locally advanced gastric cancer were enrolled between 2017 and 2021. Hypericin (Laif 900®; 900 mg) was administered orally as one single dose two to four hours prior to surgery. Standard laparoscopy was extended by ultraviolet light-laparoscopy (390-440 nm) using Storz D-Light-System. Presence and extent of peritoneal metastasis was assessed, and samples taken. Photodynamic therapy for a duration of 15 minutes was added in case of manifestations suspicious for peritoneal metastasis.

**Results**: Until 2021, 50 patients were included in the trial, among which 33 (66 %) were men. Mean age was 64.5 years. Standard and ultra-violet light-laparoscopy was feasible in all patients (100 %). There were no complications caused by study medication or intervention. CT scans showed signs for peritoneal metastasis in eight patients (16 %), among whom in six patients (75 %) diagnosis was confirmed by laparoscopy-guided biopsy. Peritoneal metastases were suspected in 26 patients (52 %) during standard laparoscopy among whom in 16 patients (64 %) diagnosis was attested by biopsy. In 25 patients (50 %) there were fluorescent areas observed by ultraviolet light examination that conformed with diagnosis of peritoneal metastasis in 62 % of cases (16/25 patients).

**Conclusion**: Hypericin-based fluorescence-enhanced laparoscopy in patients with gastric cancer was shown feasible and safe. This trial could not substantiate a clear advantage of fluorescence-enhanced laparoscopy compared to standard examination. Whether there is a clinically relevant application to enhance diagnostic value or even treating peritoneal metastasis in this way remains to be determined by larger future trials.

## ISSPP 2022 PLENARY ABSTRACT 6.

### TUMOR MICROENVIRONMENT IN PERITONEAL MESOTHELIOMA UNDER REPETITIVE PIPAC THERAPY

Lucia Eberl ^3,4^, Hannah Lee^3,4^, Marc A Reymond ^3,4^, Alfred Königsrainer^3,4^, Hans Bösmüller ^1^, Wiebke Solass ^1,2^

^1^Institute of Pathology and Neuropathology University Hospital Tübingen, Eberhard-Karls-University Tübingen, Germany; ^2^ Institute of Pathology, University Bern, Switzerland; ^3^ National Center for Pleura and Peritoneum, University of Tuebingen, Germany; ^4^ Dept. of General and Transplant Surgery, University Hospital Tübingen, Eberhard-Karls-University Tübingen, Germany

**Introduction**: Peritoneal mesothelioma is a fatal malignancy with poor prognosis and limited treatment options. A new treatment alternative is Pressurized Intraperitoneal Aerosol Chemotherapy (PIPAC). This laparoscopic drug delivery system can be repeated and allows the sequential acquisition of tumour tissue for the assessment of tumour response, but also to monitor possible changes in the tumour microenvironment (TME). We hypothesize that changes in the composition of the (TME) occur under repetitive therapy and that the changes might have an impact on the clinical outcome.

**Methods**: Within a retrospective cohort study, we selected three patients with repetitive PIPAC applications (ethical approval: 232/2022BO2). Relevant FFPE-material was selected from the archive and immunohistochemically stained with seven relevant markers (CD4, CD8, CD20, CD68, CD163, Mib1, phosSTAT). Whole slide image quantification of immune cell infiltrate was performed via Qupath, an open-source platform for bioimage analysis.

**Results**: In these three patients with peritoneal mesothelioma 1134 analyses in 133 slides were performed. In all three patients, the immune cell infiltrate differs between the TME and the central tumour area (CTA) at the beginning of the treatment. Under the course of therapy the immune cell infiltrate in both compartments change. In the TME an initial upregulation of macrophages, T-cell (CD4+ and CD8+) occur, correlating well with the morphological tumour response. In contrast to the CTA where an initial reduction of the immune cell population is observed. B-cell population and proliferation index are stable under the whole course of therapy. With progression of disease, the immune cell infiltrate is regredient to the initial state, showing an exhaustion of the immune defense.

**Conclusion**: PIPAC therapy has an impact on the composition of the TME in peritoneal mesotheliomas. The composition of the immune cell infiltrate correlates with clinical and histo-morphological tumour response. With tumour progression and exhaustion of the immune self-defense is observed, leading to a re-establishment of the initial immune landscape. Alternative treatment options like immune checkpoint blockage or other immunotherapies might overcome these treatment obstacles. Further investigation in a larger cohort of patients is needed.

## ISSPP 2022 PLENARY ABSTRACT 7.

### INFLUENCE OF THE PERITONEAL METASTATIC MICROENVIRONMENT ON INTERSTITIAL PERMEABILITY

Hooman Salavati^1-3^, Charlotte Debbaut^2-3^, Pim Pullens^4-6^, Wim Ceelen^1-3^

^1^Department of Human Structure and Repair, Ghent University, Ghent, Belgium; ^2^IBiTech– Biommeda, Ghent University, Ghent, Belgium; ^3^Cancer Research Institute Ghent (CRIG), Ghent, Belgium; ^4^Department of Radiology, University Hospital Ghent, Ghent, Belgium; ^5^Ghent Institute of Functional and Metabolic Imaging (GIFMI), Ghent University, Ghent, Belgium; ^6^IBiTech– Medisip, Ghent University, Ghent, Belgium

**Introduction**: The interstitial permeability (*k*) of solid tumor tissue correlates with the interstitial fluid pressure (IFP), and both are known determinants of drug delivery. We developed a novel method to quantify k in peritoneal metastases. Here, we report the dependency of *k* on the tumor microenvironment (TME) using quantitative histology.

**Methods**: Ten fresh ovarian peritoneal metastases were harvested, sliced, and punched into circular discs with a diameter of 12 mm and a thickness of 1 mm. We developed an apparatus based on modified Ussing diffusion chambers to evaluate the fluid transport through samples due to a hydrostatic pressure. To evaluate the dependency of k on the TME, collagen content, cell density, and fibroblast density of the samples were estimated using immune histochemistry combined with the open source software ImageJ.

**Results**: Measured *k* values (ranging between 4.8E-18 and 4.4E-17 m^2^/Pa∙s) demonstrated intra- and inter-tumoral heterogeneity of interstitium permeability, which varied up to a factor 4 in different locations of a single tumor sample. Also, significant variations of *k* were observed between different tumors of the same origin (up to one order of magnitude). These heterogeneities result in variations of IFP in solid tumors. Comparing the values of fiber, tumor cell, and fibroblast densities in different samples of a single tumor, the highest differences were found to be about 29%, 22% and 50%, respectively. The inter-tumoral analysis also showed that the variations of collagen fiber, tumor cell and fibroblast contents among different tumors can get as high as 75%, 93% and 78%, respectively. Correlating the densities of TME elements with *k* values, a significant inverse correlation was found between collagen fiber density and the corresponding *k* values (R^2^=87%, p<0.00001). Also, a moderate inverse correlation was detected between myofibroblast density and *k* values (R^2^=59%, P=0.005). In contrast, no conclusive relation was found to exist between the cell density and *k*.

**Conclusion**: The interstitial permeability of the tumor stroma is highly heterogeneous and was inversely correlated with collagen fiber density and fibroblast density.

**POSTERS OF DISTINCTION**

## ISSPP 2022 POSTER OF DISTINCTION ABSTRACT 8.

### CHARACTERIZATION OF HUMAN OMENTAL LYMPHATIC SYSTEM IN HEALTH AND CANCER

Caroline Graf^1^, Frank-Jürgen Weinreich^1^, A. Königsrainer^1,2^, G. Nadiradze^1,3^, Marc A. Reymond^1,3^, Wiebke Solass^1,3^,

^1^National Center for Pleura and Peritoneum, NCT South-West Germany, Tübingen^; 2^Dept. of General and Transplant Surgery, University Hospital, Tübingen, Germany;^; 3^Institute of Pathology and Neuropathology, University Hospital, Tübingen, Germany

**Background**: The omentum has unique lymphatic structures called milky spots (MS), making this tissue extremely attractive for immune processes, especially in terms of interactions with the peritoneal cavity. MS are the first line of defense in the early phase of peritoneal metastasis (PM) but also provide a structural and metabolic basis for the progression of PM. Due to metastatic processes, the cellular composition of the omentum changes and the expression of various chemokines that function as intercellular messengers. This study aims to gain new knowledge about the MS subpopulations, the distribution of immune cells, and the related chemokine levels in the omentum at different stages of tumor progression, which the hope of paving the way for new approaches in immunotherapy of PM.

**Methods**: The characterization of the cellular subpopulations of MS in the omentum was performed by immunohistochemistry and Fluorescence Activated Cell Sorting (FACS), whereas the chemokines in the omentum were analyzed by Enzyme-Linked Immunosorbent Assay (ELISA).

**Results**: The average cell amount in MS during the progression of PM proved to be exceptionally high in the tissue of PM patients, where the cell amount was significantly increased compared to the tissue with colon cancer without PM and the control tissue. The IHC and FACS results showed only a few cell subpopulations found throughout the omentum by both methods (0.1%- 5%). The highest numbers of M1-, M2-macrophages, and regulatory T-cells were detected in the tissue of patients with PM. The results of ELISA analysis indicated that the levels of all three tumor-promoting chemokines (CCL2, CCL22, CXCL12) increased with the progression of PM. In contrast, the tumor-inhibitory chemokine (CCL19) concentration decreased as PM progressed.

**Conclusion**: To our knowledge, this is the first detection of MS immune cell subpopulations by immunohistochemistry and FACS in human omental samples, as well as the detection of various chemokines that influence immune cells and thus the tumor environment. This opens a new observational window for characterizing the peritoneal immunological space in health and disease.

## ISSPP 2022 POSTER OF DISTINCTION ABSTRACT 9.

### OUTCOMES OF A PHASE II STUDY OF INTRAPERITONEAL PACLITAXEL PLUS SYSTEMIC CAPECITABINE AND OXALIPLATIN (XELOX) FOR GASTRIC CANCER WITH PERITONEAL METASTASES

Daryl KA Chia MRCS^1^, Jia Jun Ang MRCS^1^, Raghav Sundar MRCP ^2,3,4^, Guowei Kim FRCS^1^, Jeffrey HY Lum FRCPath^5^, Min En Nga FRCPath^5^, Giap Hean Goh FRCPath^5^, Seet Ju Ee FRCPath^5^, Cheng Ean Chee MRCP^2^, Hon Lyn Tan MRCP^2^, Jingshan Ho MRCP^2^, Natalie YL Ngoi MRCP^2^, Matilda XW Lee MRCP^2^, Vaisnavi Muthu MRCP^2^, Gloria HJ Chan MRCP^2^, Angela SL Pang MRCP^2^, Yvonne LE Ang MRCP^2^, Joan RE Choo MRCP^2^, Joline SJ Lim MRCP^2^, Jun Liang Teh FRCS^6^, Aung Lwin FRCS^6^, Yuen Soon FRCS^6^; Asim Shabbir FRCS^1,3,7^, Wei Peng Yong MRCP^2,8^, Jimmy BY So MPH, FRCS^1,3,7^

^1^Department of Surgery, University Surgical Cluster, National University Health System, Singapore; ^2^Department of Haematology-Oncology, National University Cancer Institute National University Health System, Singapore; ^3^Yong Loo Lin School of Medicine, National University of Singapore, Singapore; ^4^The N.1 Institute for Health, National University of Singapore, Singapore; ^5^Department of Pathology, National University Hospital, National University Health System, Singapore; ^6^Department of General Surgery, Ng Teng Fong General Hospital; ^7^Division of Surgical Oncology, National University Cancer Institute, National University Health System, Singapore; ^8^Cancer Science Institute of Singapore, National University of Singapore, Singapore

**Background**: Intraperitoneal paclitaxel (IP-PTX) with paclitaxel/5-fluoropyrimidine has shown promising results in patients with gastric cancer peritoneal metastases (GCPM) but has not been studied with standard-of-care platinum/fluoropyrimidine combinations. Our aim was to evaluate IP-PTX with capecitabine/oxaliplatin (XELOX) in GCPM.

**Methods**: This prospective Phase II trial recruited patients with GCPM who received IP PTX (40mg/m2,day 1,8), oral capecitabine (1000mg/m^2^ twice daily,day 1-14) and intravenous oxaliplatin (100mg/m^2^,day 1) in 21-day cycles. Patients with synchronous GCPM underwent conversion surgery if they had objective response or stable disease after chemotherapy, conversion to negative cytology taken before IP-PTX infusion, no extraperitoneal metastasis and no peritoneal disease during surgery. The primary endpoint was overall survival and secondary endpoints were progression-free survival.

**Results**: Seventy-four patients with GCPM received a median of 8 cycles of IP-PTX with XELOX (interquartile range [IQR] 5-8). The median age and Eastern Cooperative Oncology Group (ECOG) performance status was 61.5 years (IQR 56.0 – 69.0) and 1 (IQR 0 – 1).The median progression-free survival (PFS) was 9.3 months (IQR 4.9 – 14.4) and the median overall survival (OS) was 13.4 months (IQR 8.8 – 25.2). Patients undergoing conversion surgery comprised 31.3% (20/64) of patients with synchronous GCPM in the study group and had 1-year survival of 85%. The median OS of patients undergoing conversion surgery and those who did not was 25.2 months and 15.5 months respectively. (HR 0.34, 95% CI 0.18 – 0.62).

**Conclusion**: IP PTX with XELOX remains a promising treatment option for GCPM patients. In patients with good response, conversion surgery is feasible with favorable outcomes.

**POSTER PRESENTATIONS**

## ISSPP 2022 POSTER ABSTRACT 10.

### SIMULATION OF ELECTROSTATIC PRECIPITATION PRESSURIZED INTRAPERITONEAL AEROSOL CHEMOTHERAPY (EPIPAC) IN A REALISTIC HUMAN ABDOMINAL MODEL

Mohammad Rahimi-Gorji^1,2,3^, Charlotte Debbaut^2,3^, Ghader Ghorbaniasl^4^, Sarah Cosyns^1,3^, Wouter Willaert^1,3^, Wim Ceelen^1,3^

^1^Laboratory for Experimental Surgery, Department of GI Surgery, Ghent University, Ghent, Belgium; ^2^IBiTech – Biommeda, Ghent University, Ghent, Belgium; ^3^CRIG – Cancer Research Institute Ghent; ^4^Department of Mechanical Engineering, Vrije Universiteit Brussel (VUB), Brussels, Belgium

**Introduction**: Pressurized IntraPeritoneal Aerosol Chemotherapy (PIPAC) consists of a combination of laparoscopy with intraperitoneal delivery of anticancer agents as an aerosol. Adding electrostatic precipitation (ePIPAC) may improve the homogeneity of aerosols in the peritoneal cavity. In this study, we developed a computational model of (e)PIPAC based on a realistically reconstructed human peritoneal cavity.

**Methods**: An anatomically-based 3D model was generated by Mimics-Materialise using a CT dataset of the peritoneal cavity of an adult patient with a massive tension pneumoperitoneum. The resulting file was imported in COMSOL Multiphysics. Computational modelling of PIPAC consisted of filling the cavity with CO2 to reach a pressure of 12 mmHg, and nebulization of liquid under appropriate initial and boundary conditions (flow rate 0.6 mL/s, droplet diameter 30 µm, density 1020 kg/m^3^, viscosity 1 mPa·s, and freeze conditions at the walls).

**Results**:

Simulation of PIPAC alone showed that due to the effects of gravitational force and inertial impaction, aerosol droplet deposition was significantly lower at the ventral compared to the dorsal peritoneal surface (21.23% vs. 78.77%, Figure 1a). Extensive deposition of aerosol droplets was observed at the opposite site of the nebulizer. Most aerosol droplets (69.23%) were found in the central region of the peritoneal cavity (Figure 1b, regions B and C), 9.92% in the pelvic region (region A), and 20.85% in the epigastrium (region D). Adding electrostatic precipitation to PIPAC (ePIPAC) using a brush electrode (8 kV) and the whole surface of the physical model as ground (return electrode) resulted in a significant increase of deposited aerosol droplets on the ventral surface, pelvic region, and epigastrium (36.13%, 16.12%, and 25.72%, respectively) which indicated a more homogenous spatial distribution of aerosol droplets in the peritoneal cavity.

**Figure:**
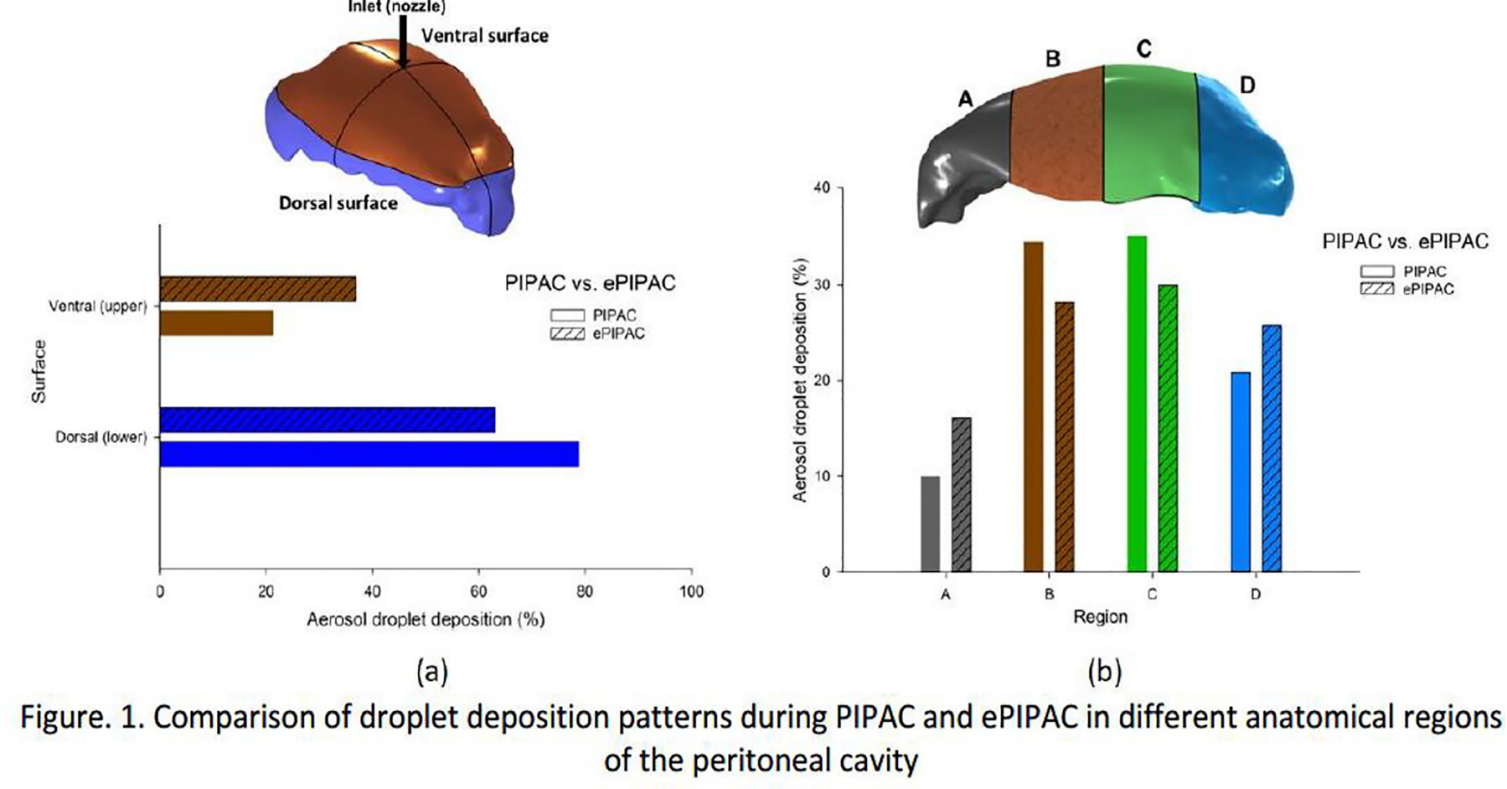

**Conclusions**: Using an electrostatic field (ePIPAC) in the reconstructed human peritoneal cavity improved the distribution of droplets, possibly leading to better anticancer efficacy.

## ISSPP 2022 POSTER ABSTRACT 11.

### INCIDENCE AND OUTCOMES OF DELAYED PRESENTATION AND SURGERY IN PERITONEAL SURFACE MALIGNANCIES

Jun Kiat Thaddaeus Tan^1^, Wong, Jolene^2^, Jane Seo^2^, Hongyuan Zhu^2^, Johnny Ong^3^, Claramae Chi^4^

^1^SingHealth Duke-NUS Academic Medical Centre; ^2^National Cancer Centre Singapore; ^3^National Cancer Centre Singapore, Sarcoma, Peritoneal and Rare Tumours; ^4^National Cancer Centre Singapore, Surgical Oncology

**Background**: Peritoneal surface malignancies (PSM) present insidiously and often pose diagnostic challenges. There is a paucity of literature quantifying the frequency and extent of therapeutic delays in PM and its impact on oncological outcomes.

**Methods**: A review of a prospectively maintained registry of PM patients undergoing Cytoreductive Surgery and Hyperthermic Intra-peritoneal Chemotherapy (CRS-HIPEC) was conducted. Causes for treatment delays were identified. We evaluate the impact of delayed presentation and treatment delays on oncological outcomes using Cox proportional hazards models.

**Results**: 319 patients underwent CRS-HIPEC over a 6-years duration. 58 patients were included in the present study. Mean duration between symptom onset and CRS-HIPEC was 186.0 ± 37.1 days (range 18-1494 days) and mean duration of between patient-reported symptom onset and initial presentation was 56.7 ± 16.8 days. Delayed presentation (> 60 days between symptom onset and presentation) was seen in 20.7% (n=12) of patients and 50.0% (n=29) experienced a significant treatment delay of > 90 days between 1^st^ presentation and CRS-HIPEC. Common causes for treatment delays were healthcare provider-related i.e. delayed or inappropriate referrals (43.1%) and delayed presentation to care (31.0%). Delayed presentation was a significantly associated with poorer disease-free survival (DFS) (HR 4.67, 95% CI 1.11-19.69, p=0.036).

**Conclusion**: Delayed presentation and treatment delays are common and may have an impact on oncological outcomes in PSM. There is an urgent need to improve patient education and streamline healthcare delivery processes in the management of PSM.

## ISSPP 2022 POSTER ABSTRACT 12.

### ELECTROSTATIC PRECIPITATION CAN SHORTEN THE TIME OF PRESSURIZED INTRAPERITONEAL AEROSOL CHEMOTHERAPY (PIPAC)

Iaroslay Sautkin^1^, Alfrd Koenigsrainer^2^, Marc Reymond^3^

^1^University Hospital Tuebingen, Department of General, Visceral and Transplant Surgery; ^2^University Hospital Tübingen, Department of General, Visceral and Transplant Surgery; ^3^University Hospital Tuebingen, Dept. of Surgery and Transplantation

**Introduction**: Clinical stidies demonstrate safety, applicability and promising histological response after PIPAC combined with electrostatic precipitation (ePIPAC). However, there is no data establishing optimal activation point and duration of electrostatitic precipitation.

**Methods**: Hypothesis: electrostatic precipitation during aerosolization can shorten PIPAC from 36min to 6min. Ex-vivo study in inverted bovine urinary bladders (eIBUB model). Aerosolization of 50ml doxorubicin (DOX) 2.7mg and 150ml cisplatin (CIS) 13.5mg. Five groups (Gr) were compared: I: electrostatic precipitation (EP) for 6min, II: EP for 10min, III: EP for 30min, IV: EP for 36min, V: control=PIPAC for 36min. Activation of EP at T0min (defined as the start of aerosolization) in groups I & IV, at T6min in groups II & III. Outcome criteria: a) aerosol tissue uptake, b) DOX tissue dosage.

**Results**: Aerosol absorption into the peritoneal tissue was superior by 177±51% in Gr I (ePIPAC, 6min vs PIPAC 36min), p>0.05. DOX tissue dosage after 6min ePIPAC was comparable with 36min PIPAC, p>0.05, and decreased with longer EP time (ng/mg: Gr I: 2.0±2.5, II: 1.3±1.1, III: 0.6±0.8, IV: 1.00±1.2, V= PIPAC (control): 2.3±1.7; (V vs II, III, IV p<0.05)). Complete DOX and CIS penetration through the bladder wall (over 4000µm) was at ePIPAC (Gr III and IV).

Aerosol absorption was superior after 6min ePIPAC. DOX tissue dosage after 6min ePIPAC (starting at T0) approaches PIPAC after 36min. DOX tissue dosage decreased over time, suggesting the electrostatic field transporting the drug to the outer eIBUB surface after initial tissue uptake.

**Conclusion**: Electrostatic precipitation should be activated at the time of aerosolization. ePIPAC

for 6min is not inferior to PIPAC 36min.

## ISSPP 2022 POSTER ABSTRACT 13.

### PATIENT REPORTED OUTCOMES AFTER PRESSURIZED INTRA-PERITONEAL AEROSOLIZED CHEMOTHERAPY (PIPAC) IN THE PALLIATION OF PATIENTS WITH UNRESECTABLE PERITONEAL METASTASES

Jolene Wong, Jolene^1^, Jane Seo^1^, Darryl Juan^1^, Johnny Ong^2^, Claramae Chia^3^

^1^Singapore General Hospital; ^2^National Cancer Centre Singapore, Sarcoma, Peritoneal and Rare Tumours; ^3^National Cancer Centre Singapore, Surgical Oncology

**Introduction**: Patient with unresectable peritoneal metastases (PM) represent a clinical challenge as current strategies for symptom palliation are largely ineffective due to the poor penetration of systemic agents into the peritoneal cavity. Pressurized Intra-Peritoneal Aerosolized Chemotherapy (PIPAC) boosting improved drug delivery via direct and repeatable intra-peritoneal (IP) application is a potential modality that can be used to improve palliative outcomes for these patients.

**Methods**: We conduct a single arm prospective clinical trial aimed at the assessing the effectiveness of PIPAC as a mode of palliation for local symptoms in patients with unresectable PM. Palliative outcomes were assessed using health related quality of life scores (HRQoL): European Organization for Research & Cancer Treatment EORTC-QLQ C30, Functional Assessment of Cancer Therapy-General (FACT-G) and Integrated Palliative Outcome Scale (IPOS). Questionnaires were administered at baseline, and after consecutive PIPAC sessions.

**Results**: A total of 28 patients underwent 63 PIPAC procedures over a 1-year duration. Mean number of PIPACs per patient was 2.3 (range 1 to 4). 44% had primary colorectal tumors, 16% upper gastro-intestinal, 6% hepatobiliary, 5% appendiceal, 5% mesothelioma, 24% other rare origins (including breast, gynecological, small bowel, unknown sites). Median PCI score was 17.1 at baseline, and it was 19, and 17.6 prior to the 2^nd^ and 3^rd^ PIPACs respectively. There were no serious adverse events and patient, and mean length of hospitalization was 2 days. Global-QoL Score saw a significant increase from a mean of 56.7 to 81.3 after consecutive PIPACs; a similar trend was seen in functional scores-physical which improved from 72.7 to 87.5 points. GI-specific symptom scores also a more than 2-fold decline. A similar trajectory was seen in both the FACT-G and IPOS scores.

**Conclusion**: Consecutive PIPACs resulted in significant improvements in HRQoL amongst patients with unresectable PM and should be considered as a treatment modality for the palliation of PM symptoms.

## ISSPP 2022 POSTER ABSTRACT 14.

### EFFECT OF ELECTROMOTIVE DRUG ADMINISTRATION ON PERITONEAL TISSUE PENETRATION OF NANOPARTICLES

Wong Si Min Jolene^,1^, Nidda Saeed^2^, Jesse Demuytere^3^, Annelies Coene^4^, Wim Ceelen^5^

^1^National Cancer Centre, Singapore; ^2^Ghent University, Laboratory of Experimental Surgery; ^3^UGent, Structure and repair; ^4^Ghent University; ^5^Ghent University, Surgery

**Introduction**: Intraperitoneal (IP) drug delivery for the treatment of peritoneal metastases is often limited by high intra-tumoral pressures and the relative impermeability of the peritoneal membrane. The use of an electromotive force to enhance drug transport has been adopted in a wide variety of clinical conditions including the treatment of bladder and skin cancer. We hypothesize that electromotive drug administration (EMDA) using a pulsed DC current can enhance IP drug penetration and distribution. In this study, we aimed to establish a novel peritoneal-specific EMDA model and examine its impact on penetration of nanoparticles (NPs) in peritoneal tissue and define the optimal conditions for drug transport.

**Methods**: An EMDA-peritoneal experimental model was devised and comprised of a partially insulated apparatus connected to a current generator *(Physionizer® Mini 30N2).* Fluorescent nanoparticles (200nm NP, amine cationic *FluoSpheres* ^*TM*^) suspended in carrier solutions were used to evaluate the impact of EMDA on tissue penetration and spatial distribution in porcine peritoneal tissue. We performed a series of experiments to evaluate the use of varying current amplitude, treatment duration, carrier solution, temperature, and pressure during EMDA and its effect on NP penetration.

**Results**: EMDA resulted in a 4-fold increase in penetration and a 2-fold improvement in spatial distribution compared to passive diffusion (p=0.0006). A significant positive correlation between current amplitude and NP penetration was found (r=0.8). The optimal duration of EMDA treatment was 40 minutes, and longer exposure times did not result in increased NP delivery. The performance of NPs within isotonic solutions (peritoneal dialysate and 0.9% NaCl) was optimal and resulted in enhanced penetration compared to hypotonic and hypertonic carriers. When combined with hyperthermia, NP penetration was found to be significantly improved over all other test groups (p=0.0001)

**Conclusion**: EMDA has the potential to improve NP tissue penetration during intraperitoneal drug delivery.

## ISSPP 2022 POSTER ABSTRACT 15.

### ROLE OF DIVERTING OSTOMY WITH MULTIPLE INTESTINAL ANASTOMOSES AFTER HIPEC: A PROPENSITY SCORE MATCHING STUDY

Amaniel Kefleyesus^1,5^, Vahan Kepenekian^1^, Isabelle Bonnefoy^1^, Gonzalo Guinez^2^, Olivia Sgarbura^2^, Barbara Noiret^3^, Clarisse Eveno^3^, Aaron Fernandes^4^, SP Somashekhar^4^, Daniel Clerc^5^, Hugo Teixeira-Farinha^5^, Martin Hübner^5^, Olivier Glehen^1^

^1^Department of Surgical Oncology, Lyon University Hospital, Centre Hospitalier Lyon-Sud, Lyon, France; ^2^Department of Surgical Oncology, Cancer Institute Montpellier (ICM); ^3^University of Montpellier, Montpellier, France; ^4^Department of Digestive and Oncological Surgery Claude Huriez University Hospital, Lille, France; ^5^Department of Surgical Oncology, Manipal Comprehensive Cancer Center, Bengaluru, India; ^6^Department of Visceral Surgery, Lausanne University Hospital CHUV, Lausanne, Switzerland.

**Background**: Performing multiple intestinal anastomoses, including rectal anastomoses after CRS-HIPEC appears to be safe. However gastrointestinal leak (GIL) after CRS-HIPEC is associated with significant morbidity and mortality. Number of anastomoses including small bowel, large bowel, and combination of them are risk factors for GIL. Impact of ostomy creation in CRS-HIPEC patients was associated with poor outcomes regarding recurrence and potential subsequent morbidity. Few data are available regarding those outcomes associated with ostomy creation after multiple large bowel anastomosis for colorectal peritoneal metastases (PM) in patients undergoing curative intent CRS-HIPEC. This multicentre retrospective study aimed to assess the ostomy impact as predictors of survival and morbidity in a homogenous cohort with a propensity score matching study (PSM).

**Methods**: A PSM study with multicenter retrospective data of consecutive patients curatively treated with CRS-HIPEC only for colorectal PM etiology. All patients had at least 2 large bowel resections with primary anastomoses with(WO) or with no (NO) diverting ostomy. Survival and morbidity predictors were described with hazard ratios and odds ratios.

**Results**: A total of 183 patients were included. After a 1: 2 PSM, we performed comparative analyses between NO (n=45) and WO (n=95) patients. Patients were comparable for the main covariates (gender, age, PCI, comorbidity, past surgical history, resected organs). NO patients had more anastomotic leak (AL) (15.6 vs 6.7%, p=0.1), shorter median hospital stay (14 vs 21 days, p < 0.01), but with comparable severe complications rate (Clavien >3a) (24.4 vs 25.6%, p=0.9) and 90-days mortality (2.2 vs 0%, p=0.16). Ostomy was not associated as predictor factor in overall and recurrence-free survival analyses. However, multivariate analyses showed WO patients as independent risk factor of mortality (OR 3.7, p = 0.009) and recurrence (OR 3.1, p = 0.01) adjusted to the main covariates including PCI and severe complications.

**Conclusion**: Several data are suggesting the protective ileostomy not to be required for single anastomosis after CRS-HIPEC thus a diverting ileostomy for 2 large bowel anastomoses might be not necessary regarding this study. Further prospective studies might confirm our findings.

## ISSPP 2022 POSTER ABSTRACT 16.

### PROGNOSTIC IMPACT OF SIGNET RING CELL PROPORTION IN COLORECTAL CANCER PATIENTS WITH PERITONEAL METASTASES

Vahan Kepenekian^1^, Amaniel Kefleyesus^2^, George Petrides^3^, Shoma Barat^4^, Isabelle Bonnefoy^5^, Jullien Peron^6^, Olivier Glehen^7^, David Morris^8^

^1^Centre Hospitalier Lyon-Sud, Surgical Oncology; Université Claude Bernard Lyon 1, CICLY EA3738; ^2^Centre Hospitalier Universitaire de Lyon, Department of Surgery, Pierre-Bénite; Lausanne University Hospital, Department of Visceral Surgery; ^3^University of New South Wales Faculty of Medicine Valle, Sarah; ^4^Saint George Hospital, Department of Surgery, Peritonectomy Unit; University of New South Wales - Saint George Campus; ^5^University Hospital Centre Lyon, Department of Clinical Research; ^6^Hospital, Department of Surgery, Peritonectomy Unit; University Hospital Centre Lyon, Surgical Oncology Department - CICLY EA3638; Université Claude Bernard Lyon 1, CICLY EA36738; ^8^University of New South Wales, St George Hospital, Faculty of Medicine, Department of Surgery

**Introduction**: Rarely, colorectal cancers (CRC) comprise signet ring cells (SRC) made of cytoplasmic mucin and responsible for a poor prognosis. The classification considers a primary tumor as a SRC tumor when the SRC component (SRCc) represent more than half of the cells. However, the prognostic impact of lower SRCc in CRC patients with peritoneal metastases (pmCRC), candidate for cytoreductive surgery, remains unclear, jeopardizing a proper selection.

**Methods**: A retrospective analysis of a bicentric database, both prospectively maintained, was performed to assess the impact of the SRC proportion by comparison to classical adenocarcinoma (cADK) (non SRC and non-mucinous). Appendiceal cancers were excluded. The SRC proportion was reviewed by an expert pathologist. Uni/multivariate survival analyses with Cox model regression were conducted. Missing data was dealt with imputation. Survival rates were estimated using the Kaplan-Meier method and compared with the log-rank test.

**Figure 1:**
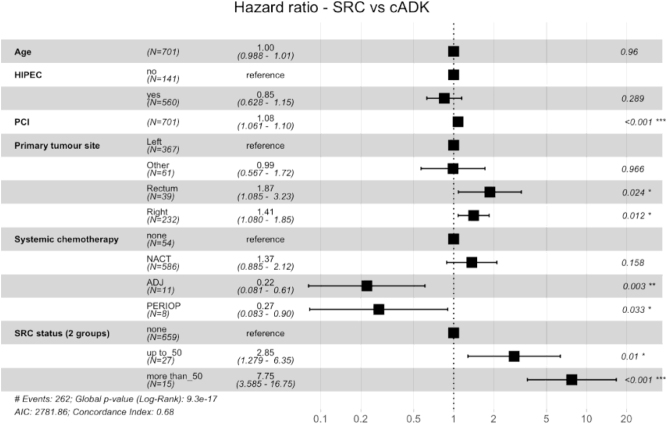
Multivariate analysis showing harzard ratio between SRC vs cADK.

**Results:** Overall, 59 pmCRC patients with a SRCc were included (15 patients with SRCc >50%) and compared to 647 cADK patients with peritoneal metastases. Thirty-seven patients (63%) had a complete resection, all but one followed by Hyperthermic IntraPeritoneal Chemotherapy. Except one patient, all patients had systemic chemotherapy, perioperatively in 46% of cases. When compared to cADK, the SRC patients were younger, more often with right-sided tumors, with pN2 status and with higher peritoneal carcinomatosis index (median 16.0 *vs* 8.6, respectively). After a median follow-up of 40 months [IC95%, 36-45], the median overall survival was 16, 20 and 42 months in patients with ≤50% SRC, >50% SRC and cADK respectively (p<0.001). In multivariate analysis SRCc ≤50% (HR1.81, [IC95%, 1.11-2.97], p<0.018) and SRCc >50% (HR2.09, [1.35-2.26], p<0.001) (Figure 1) Incomplete cytoreduction and severe postoperative complications were associated with a poorer survival. After matching 3:1, lymph node invasion, PCI>15 and SRCc >50% (HR2.11, [1.25-3.60], p=0.005) were predictive of poor survival.

**Conclusion**: A component of SRC in pmCRC patients candidate for cytoreduction appeared as a strong negative prognosis factor, even when the SRCc was lower than 50%.

## ISSPP 2022 POSTER ABSTRACT 17.

### POST-HOC ANALYSIS TO IDENTIFY REASONS TO STOP PRESSURIZED INTRAPERITONEAL AEROSOL CHEMOTHERAPY (PIPAC) BEFORE THE 3 RECOMMENDED FOR IMPROVING FUTURE PATIENT SELECTION

Anne-Cecile Ezanno^1^, Brice Malgras ^1^, Jade Fawaz ^2^, Adeline Aimé ^1^, Hugo Picchi ^3^, Solène Doat^4^, Marc Poacrd^2^

^1^Service de Chirurgie HIA Begin - Saint-Mandé (France), ^2^Service de Chirurgie La Pitié Salpétrière, Paris^,^ France; ^3^Service D’oncologie HIA Begin, St Mandé, France; ^4^Service d’oncologie Digestive La Pitié Sapétrière, Paris, France

**Introduction**: To improve patients’ prognosis and protect quality of life in case of peritoneal metastasis (PM), news treatment emerged as Pressurized intraperitoneal aerosol chemotherapy (PIPAC). Proposed in the management of several cancers, the current recommendations foresee at least 3 PIPAC. Although repetitive PIPAC is feasible in most patients, many patients have to stop after only 1 or 2 procedures. The present study analyzes causes that led to the stop PIPAC.

**Methods**: Prospective multicenter cohort study included all patients with PM undergoing PIPAC in the 3 PIPAC expert centers between 2015 and 2021.

**Results**: A Total of 268 PIPAC procedures were performed in 89 patients. The origins of the PM were colorectal, gastric, ovarian, mesothelioma and biliopancreatic. 48.3% patients underwent less than 3 PIPAC: 28.1% had one PIPAC, 20.2% two PIPAC and 51.7% had three or more. The main reason to stop PIPAC whatever the number of procedures is disease progression 55.8%. Other reasons to stop PIPAC were non-access to abdominal cavity in 7.9%, conversion to cytoreductive surgery in 13.5%, adverse event post PIPAC in 7.9% (intra-operative bowel injury, bowel obstruction, bleeding, or healing difficulty), patients’ wish in 10.1% and death in 2.2%. In subgroup analyze, patients with<3 PIPAC, disease progression and adverse event were always the main reasons to stop PIPAC. Only adverse event was significatively (p= 0.05). The analysis of patients who received less than 3 PIPACs showed patients that patients had less frequent chemotherapy before (91% vs. 100%, p=0.05), more bimodal treatment (40% vs. 30%, p=0.048), more ascites (1116ml± 2137 vs. 474±1559, p=0.05) and more often carcinomatosis ascites (73% vs. 39%, p=0.017)

**Conclusions**: Performing PIPAC alone in chemotherapy-naïve patients with ascites seems to be a treatment option to be discarded.

## ISSPP 2022 POSTER ABSTRACT 18.

### EARLY EVIDENCE FOR THE IMMUNOMODULATORY ROLE OF PARACRINE FACTORS VIA PARACRINE SIGNALLING IN COLORECTAL PERITONEAL CARCINOMATOSIS

Qiu Xuan Tan^1,2,3^, Sasinthiran Thiagarajanw^1,2,3^, Hui Jun Lim^l,^2,3, Joey Wee-Shan Tan^l,2,3^, Josephine Hendrilcson^1,2,3^, Gillian Ne^1,2,3^, Ying Lite^1,2,3^, Wai Har Ne^1,2,3^, Clara Yieh Lin Chong^1,2,3^, Xing-Yi Sarah Ong^l,2,3^, Chin Jin Seo^2,3^, Jolene Si Min Wong^2,3^, Claramae Shulyn Chia^2,3^, Chin-Ann Johnny Ong^1,2,3^

^1^ Laboratory of Applied Human Genetics, Division of Medical Sciences, National Cancer Centre Singapore, Singapore 169610, Singapore; ^2^ Department of Sarcoma, Peritoneal and Rare Tumours (SPRinT), Division of Surgery and Surgical Oncology, National Cancer Centre Singapore, 11 Hospital Cresent, Singapore 169610, Singapore; ^3^ Department of Sarcoma, Peritoneal and Rare Tumours (SPRinT), Division of Surgery and Surgical Oncology, Singapore General Hospital, Singapore 169608, Singapore

**Introduction**: Ascites is one of the late manifestations in peritoneal carcinomatosis (PC) patients. Few studies have assessed the cross talk between the cellular tumour-stroma composite and fluid microenvironment. We postulate that the paracrine-rich fluid microenvironment plays a critical role in tumorigenesis and tumour metastasis.

**Methods**: Malignant and benign ascites collected intraoperatively from patients with colorectal PC (n=3) and benign serous cystadenofibroma (n=1) was subjected to mass spectrometry. Proteomics analysis was performed to identify key paracrine factors within the fluid microenvironment that drives tumourigenesis and metastasis in CPC. Target validation was performed in ascites of PC patients (n=39) using enzyme-linked immunosorbent assay (ELISA). Clinical relevance was assessed via immunohistochemical staining on tissue microarray of primary CRC tumours (n=210).

**Results**: We identified 3627 protein targets within the ascites of CPC patients. By applying the DAVID bioinformatics tool, the list of proteins was systematically condensed to 627 candidates with high confidence. The top 10% upregulated putative markers were then manually curated via literature evidence to assess for their biological functions and relevance in cancer therapeutics. Notably, VSIG4, a coinhibitory ligand that inhibits T-cell activation, was selected for downstream evaluation of its immunomodulatory effect in CPC, which could be harnessed for immunotherapy if proven successful. Target validation using ELISA demonstrated varying levels of VSIG4 upregulation across ascites of colorectal origin, suggesting potential biological significance among the patients with enriched VSIG4 levels in ascites. Additionally, low VSIG4 expression in stroma of primary CRC is associated with poor prognosis [p= 0.018], highlighting the clinical relevance of dysregulated VSIG4 in PC.

**Conclusions**: VSIG4 secreted into the paracrine fluid microenvironment may confer an immune modulatory phenotype in patients with PC.

## ISSPP 2022 POSTER ABSTRACT 19.

### SAFETY OF CYTOREDUCTIVE SURGERY WITH HEATED INTRAPERITONEAL GEMCITABINE AND SYSTEMIC DACARBAZINE FOR RECURRENT UTERINE LEIOMYOSARCOMA – PHASE 2 TRIAL

Beatrice Sun^1^, Deshka Foster^1^, Renz, Malte^2^, Oliver Dorigo^2^, Amer Karam^2^, Nam Bui^3^; Kristen Ganjoo^3^, Byrne Lee^1^

^1^Stanford University School of Medicine, Department of Surgery; ^2^Stanford University School of Medicine, Department of Gynecologic Oncology; ^3^ Stanford University School of Medicine, Department of Medical Oncology

**Introduction**: Uterine Leiomyosarcoma (ULMS) is a rare, aggressive malignancy with high rates of recurrence and poor survival. Cytoreductive surgery (CRS) with systemic gemcitabine and docetaxel are the mainstay of treatment. Recurrence is often local in the peritoneal cavity due to rupture of the original tumor or in some cases, surgical morcellation. This prospective phase 2 study aims to investigate the effect of Heated Intraperitoneal Chemotherapy (HIPEC) using gemcitabine followed by systemic dacarbazine after optimal cytoreduction.

**Methods**: Patients with recurrent ULMS in the peritoneum deemed resectable were evaluated for inclusion. Blood for circulating tumor DNA and other correlative studies was collected pre and post operatively. After optimal cytoreduction, HIPEC with gemcitabine 1000mg/m2 was perfused for 60 minutes at 42°C. Following recovery, 6 cycles of systemic dacarbazine 1000mg/m2 was given every three weeks. Patients are then followed with cross sectional imaging every three months. Primary endpoint will be progression free survival. Secondary endpoints will be safety and quality of life correlates.

**Results**: As of July 2022, 10 patients have been enrolled. 5 patients had a history of specimen morcellation. Median peritoneal cancer index (PCI) was 7.5. All patients had a completeness of cytoreduction (CCR) score of 0 or 1. Median length of stay is 6.5 days. There have been no operative mortalities, 3 patients had a complication related to CRS and HIPEC (bladder injury, ureteral transection, severe neutropenia). Treatment related adverse events grade 3 or higher were reported in 2 patients, all were related to laboratory abnormalities.

**Conclusion**: Early results of our study show the treatment of recurrent ULMS with CRS and HIPEC using gemcitabine followed by adjuvant dacarbazine is safe and well tolerated. Further accrual and analysis will be necessary to report on the effects on the primary endpoint.

## ISSPP 2022 POSTER ABSTRACT 20.

### EVALUATION OF ALTERNATIVE THERAPEUTIC REGIMENS APPLIED AS PIPAC: STATUS QUO AND EVIDENCE. ALTERNAT-IP STUDY

Manuela Robella^1^, Martin Hubner^2^, Naoual Bakrin^3^, Aditi Bhatt^4^, Abdelkader Taibi^5^, Hugo Teixeira^6^, Wouter Willaert^7^, Andrea DiGiorgio^8^, Olivia Sgarbura^9^

^1^Candiolo Cancer Institute, Unit of Surgical Oncology, ^2^Lausanne University Hospital CHUV, University of Lausanne, Department of Visceral Surgery, ^3^Centre Hospitalier Lyon-Sud, Surgical Oncology; ^4^Fortis Hospital, Surgical Oncology; ^5^Department of Digestive Surgery, CHU Dupuyren. Somashekhar, S.P.; Manipal Comprehensive Cancer Center, Manipal Hospital, Bangalore, India., Department of Surgical Oncology, Teixeira Farinha, Hugo; ^6^Lausanne University Hospital CHUV, University of Lausanne, Department of Visceral Surgery; ^7^Department of GI Surgery and Cancer Research Institute Ghent (CRIG), ^8^Ghent University Hospital Policlinico Universitario Agostino Gemelli IRCCS, Peritoneum and Retroperitoneum Surgical Unit; ^9^Regional Cancer Research Institute Montpellier, Surgical Oncology

**Introduction**: Pressurized intraperitoneal aerosol chemotherapy (PIPAC) is a treatment option for patients with peritoneal surface malignancies of different origin and a considerable research effort was dedicated to the analysis of its initial results. The drug combinations currently used and approved are PIPAC-DC (doxorubicin 2.1 mg/sm and cisplatin 10.5mg/sm) and PIPAC-Ox (oxaliplatin 92 mg/sm). While there is a consensus about the routine use of these drug regimens, some patients may be resistant to current combinations. Some of the expert centers punctually applied alternative regimens to respond to these personalized patient need. The aim of the present study (Alternat-IP) is to assess the feasibility and safety of the current PIPAC alternative regimens in international centers.

**Methods**: All the centers identified to use alternative PIPAC drugs at the PIPAC survey were invited to participate in this retrospective study. Out of the 13 identified centers, nine accepted. All cases of PIPAC performed with mitomycin C, irinotecan or any other alternative drugs and/or combinations were. The primary objective was the safety profile of these empirical regimens.

**Results**: The nine international expert centers applying alternative PIPAC drug regimens are located in five different countries (Italy, France, Belgium, Switzerland and India). Twenty-four cases were submitted to alternative treatment (primary peritoneal cancer n=1; colorectal cancer n=10; appendiceal cancer n = 4; ovarian cancer n=3; gastric cancer n=4; pancreatic cancer n=2). A total of 80 PIPAC procedures were included. Fourteen patients were submitted to PIPAC with mitomycin C (10 patients at a dose of 1.5 mg/sm, 2 patients at 14 mg/sm and 1 at 4 mg/sm) for a total of 43 PIPAC procedures. No major complications were reported; 5 CTCAE grade 2 complications were described (nausea n =2; abdominal pain n= 1; fever n = 1). No toxicities were recorded for PIPAC with Irinotecan at a dose of 20 mg/sm (n = 3) or 30 mg/sm (n=2), for patients with Nab-paclitaxel 112.5 mg/sm (n=3), nor for patients with docetaxel 10 mg/sm + cisplatin 7.5 mg/sm + doxorubicin 1.5 mg/sm (n=2). Two patients were submitted to PIPAC with Paclitaxel 10 mg/sm (5 PIPAC in total) with one CTCAE grade 3 complication (bowel fistula).

**Conclusions**: Alternat-IP study allowed us to explore the different alternative drug regimens. PIPAC with mytomicin C 1.5-4 mg/sm is safe. The use of irinotecan and nab-paclitaxel is feasible and safe. Feasibility and safety of paclitaxel and the triplet of docetaxel+cisplatin+doxorubicin must be confirmed by wider case histories. The results obtained could represent a valid starting point for new phase I studies.

## ISSPP 2022 POSTER ABSTRACT 21.

### C-REACTIVE PROTEIN ALBUMIN RATIO AS A PREDICTOR OF MAJOR MORBIDITY AFTER CYTOREDUCTIVE SURGERY AND HYPERTHERMIC INTRAPERITONEAL CHEMOTHERAPY (CRS/HIPEC) IN OVARIAN CANCER

Luis Felipe Falla Zuniga^1^, Mary Caitlin King^1^, Teresa Diaz-Montes^2^, Felipe Lopez-Ramirez^1^, Andrei Nikiforchin^3^, Phillip Barakat^1^, Armando Sardi^1^, Vadim Gushchin^1^

^1^Mercy Medical Center; ^2^Surgical Oncology, Mercy Medical Center; ^3^The Institute for Cancer Care, Mercy Medical Center

**Introduction**: Combination serum inflammatory/nutritional markers, as opposed to single labs or complicated indexes, have consistently shown to provide useful and easily obtainable information to predict postoperative outcomes in various malignancies. However, it has not been explored with cytoreductive surgery with hyperthermic intraperitoneal chemotherapy (CRS/HIPEC), which poses significant risks for morbidity. We evaluated the predictive value of preoperative inflammatory/nutritional markers on major postoperative morbidity after CRS/HIPEC in patients with advanced ovarian cancer (OC).

**Methods**: A single-center retrospective cohort study (2000-2022) was performed. Females who underwent optimal (CC0/1) CRS/HIPEC for peritoneal metastases from OC were identified. Clavien-Dindo 90-day major (grade III/IV) complications (MC) were recorded. The ability of preoperative serum markers (C-reactive protein [CRP], albumin, CRP: albumin ratio [CAR], prealbumin, neutrophil: lymphocyte ratio, platelet: lymphocyte ratio, Glasgow prognostic score, and CA-125) to predict MC and cutoffs was determined with area under the receiver operating characteristic curve (AUC) analysis. Factors associated with elevated markers were analyzed.

**Results**: Of 172 patients, 47 (27.3%) had MC. CAR, prealbumin, and CA-125 showed the highest predictive ability for MC. CAR had the highest AUC (0.65 vs. 0.63 vs. 0.60) with a cutoff of 0.15 (sensitivity: 70%, specificity: 54%). Of 130 patients, 69 (53.1%) had elevated CAR (≥0.15). Fewer patients with elevated CAR underwent prior chemotherapy (59.6% vs 78.4%, p=0.02) or surgery (37.7% vs 59.0%, p=0.02), but had higher estimated blood loss during CRS/HIPEC (median: 850 [IQR: 500-1500] vs 500 [IQR: 350-700] mL, p<0.01). No difference in tumor burden or other intraoperative variables was observed.

**Conclusions**: Preoperative inflammation and poor nutritional state, assessed by CAR, showed predictive value for MC after CRS/HIPEC in OC patients and was independent of tumor burden. Identifying high-risk patients for MC could help guide pre-habilitation before CRS/HIPEC and decrease morbidity.

## ISSPP 2022 POSTER ABSTRACT 22.

### EARLY POSTOPERATIVE HEMATOLOGIC TOXICITY AFTER HYPERTHERMIC INTRAPERITONEAL CHEMOTHERAPY

Felipe Lopex-Ramirez^1^; Vadim Gushchin^2^ Mary Caitlin King^2^; Teresa Diaz-Montes^2^; Luis Felipe Falla Zuniga^2^; Phillip Barakat^2^; Andrei Nikiforchin^3^; Carol Nieroda^2^; Armando Sardi^2^; Panayotis Ledakis^2^

^1^Mercy Medical Center, Department of Surgical Oncology; ^2^Mercy Medical Center; ^3^The Institute for Cancer Care, Mercy Medical Center

**Introduction**: Despite localized chemoperfusion during cytoreduction with hyperthermic intraperitoneal chemotherapy (CRS/HIPEC), absorption through the peritoneum results in poorly defined systemic effects that vary by agent. We compared early postoperative hematologic toxicity (HT) of common HIPEC agents after CRS/HIPEC.

**Methods**: A retrospective cohort study was performed including patients who underwent CRS/HIPEC (2013-2021) with mitomycin-C (MMC), melphalan, or carboplatin (CARB). Daily postoperative white blood cell (WBC) and platelet (PLT) levels were obtained. Nadir values were used to assess HT-grades (G) according to CTCAE v5.0. The association between HT-G and 90-day major Clavien-Dindo complications and mortality was determined with multivariable logistic regression, adjusted by age and tumor burden.

**Results**: Of 512 included cases, 291 (56.8%) were perfused with MMC, 109 (21.3%) with melphalan, and 112 (21.9%) with CARB. Median hospital stay was 9 (IQR: 8–12) days. Major complications occurred in 104 (20.3%) cases and 12 (2.3%) postoperative mortalities occurred. After perfusion with MMC, melphalan, and CARB, 200 (68.7%), 41 (37.6%), and 66 (58.9%) cases had no WBC toxicity, respectively (p<0.01). G1-2 WBC toxicity (<4.5–2.0 x10^9^/L) occurred in 79 (27.1%), 36 (33.0%), and 31 (27.7%) (p=0.14) and G3-4 (<2.0 x10^9^/L) occurred in 12 (4.1%), 32 (29.4%), and 15 (13.4%) (p<0.01), respectively. No PLT toxicity was seen in 142 (48.8%), 33 (30.3%), and 39 (34.8%) cases, respectively (p<0.01). G1-2 PLT toxicity (<150–50 x10^9^/L) occurred in 147 (50.5%), 46 (42.2%), and 42 (37.5%) (p=0.37) and G3-4 (<50 x10^9^/L) occurred in 2 (0.7%), 30 (27.5%), and 31 (27.7%) (p<0.01). G3-4 WBC and G3-4 PLT toxicity were associated with major complications (WBC: OR=2.1, p=0.02; PLT: OR=2.2, p<0.01) and 90-day mortality (WBC: OR=6.2, p<0.01; PLT: OR=8.2, p=0.01). The hematologic toxicities are depicted in Figure 1.

**Figure:**
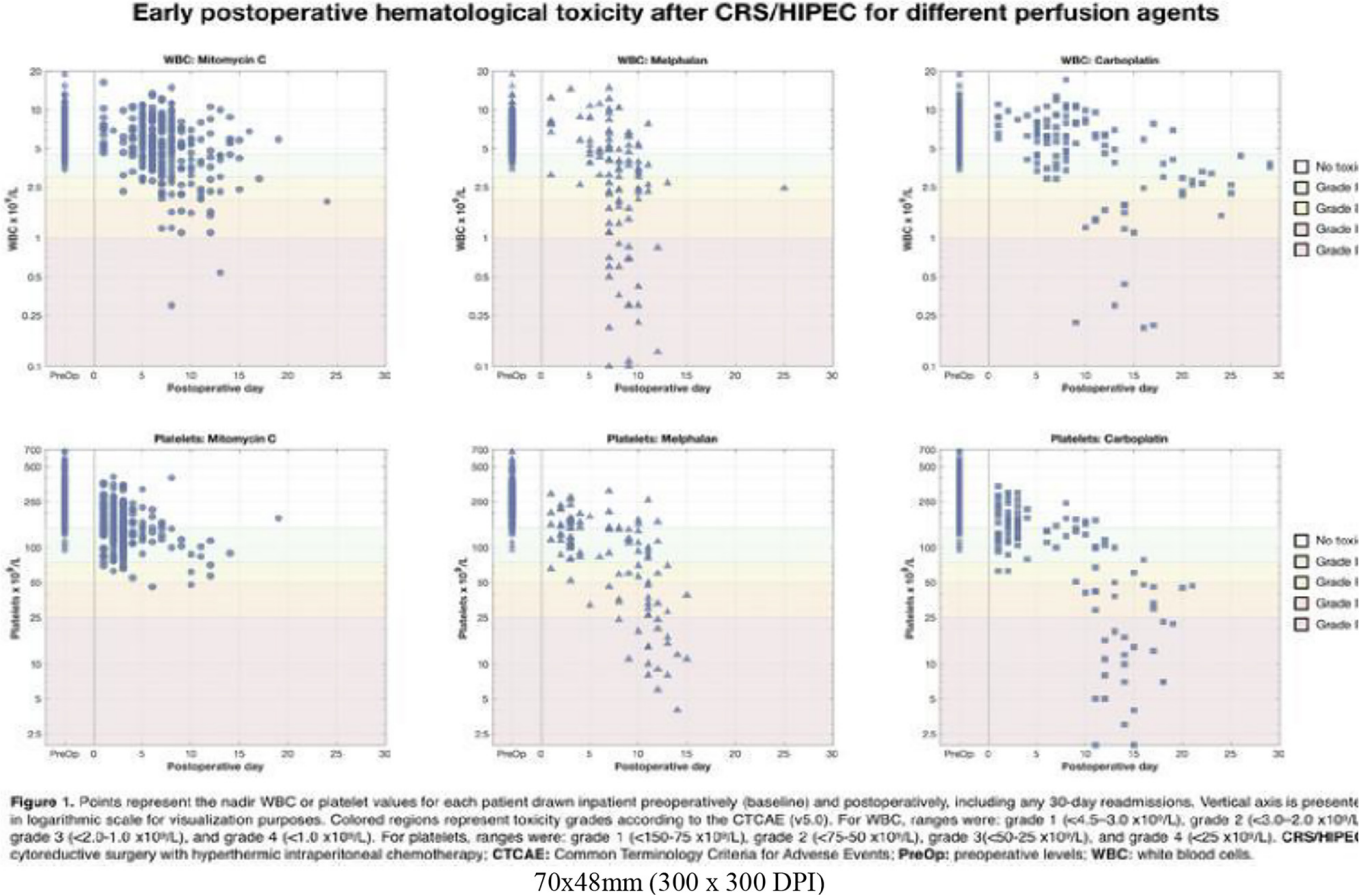


**Conclusions**: Melphalan had significantly greater WBC toxicity than MMC or CARB. PLT toxicity was similar for melphalan and CARB, which was significantly greater than MMC. Understanding HIPEC related HT and identifying patients at higher risk could aid in agent selection and early interventions to improve the safety profile of CRS/HIPEC.

## ISSPP 2022 POSTER ABSTRACT 23.

### CYTOREDUCTIVE SURGERY AND HIPEC FOR MALIGNANT MESOTHELIOMA: OUTCOMES AND SURVIVAL FROM AN AUSTRALIAN CENTER

Raymond Hayler MD, MS^1,2^, Raphael Shamavonian, MBBS^1,3^, Ernest Cheng, MD, MS^1,2^, Josh B. Karpes, MBBS, MS^1,2^, Shoma Barat, BSc, MHIM^1,2^, Nima Ahmadi, MBBS, MS, FRACS, ^1,2,^ David L. Morris, MB, ChB, FRCS, MD, PhD^1,2^ *

^1^Hepatobilliary and Surgical Oncology Unit, Department of Surgery, St George Hospital, Kogarah, NSW, Australia; ^2^St George and Sutherland Clinical School, University of New South Wales, Sydney, NSW, Australia; ^3^School of Medicine, University of Notre Dame, Sydney, NSW, Australia

**Disclosures**: There are no relevant disclosures for this research. Outside the scope of this abstract, Professor David Morris is the co-inventor and director of the company Mucpharm Pty Ltd, unrelated to this project.

**Background/Aim**: To determine outcomes and overall survival (OS) in patients undergoing cytoreductive surgery (CRS) and heated intraperitoneal chemotherapy (HIPEC) for malignant peritoneal mesothelioma (MPM).

**Methods**: Retrospective cohort study from a prospectively maintained database of patients that underwent CRS/HIPEC for MPM from April 1999 to December 2021.

**Results**: 81 patients were identified with MPM. Median OS was 53 months with a 1-, 3- and 5- year OS of 76%, 55% and 49% respectively. Multivariate analysis identified lymph node status, PCI and CC score as statistically significant prognostic factors that impact survival. Median OS for PCI 0 – 20 was 103 months vs 33 months for PCI 21 – 39 (P = 0.005). Median OS for CC0, CC1 and CC2 were 104, 30 and 2.7 months respectively (P < 0.001). Hazard ratio for node positive disease over node negative was 2.14 (95% CI 1.07 – 4.31, P < 0.033). Grade III/IV complication rate was 43.2% and mortality 4.9%.

**Conclusion**: CRS/HIPEC remains the gold standard for treating patients with MPM with excellent patient OS. Lymph node status, PCI and CC score were independent prognostic factors that affect OS.

## ISSPP 2022 POSTER ABSTRACT 24.

### ELECTROSTATIC PRECIPITATION PREVENTS THE ENLARGEMENT OF AEROSOL PARTICLES DURING PIPAC

Iaroslav Sautkin^1^, Alfred Koenigsrainer^2^, Reymond, Marc^2^

^1^University Hospital Tübingen, Department of General, Visceral and Transplant Surgery;^2^University Hospital Tuebingen, Dept. of Surgery and Transplantation

**Introduction**: Intraperitoneal liquid chemotherapy has inferior drug distribution to aerosol one. In our experience, liquid collecting overtime during PIPAC. We assume that fine aerosol particles can get larger by colliding, leading to liquid formation. The efficiency of aerosol chemotherapy might be improved by precipitation of the fine fraction, preventing further enlargement.

**Methods**: Hypothesis (HP): in PIPAC 1) electrostatic precipitation (ePIPAC) can prevent the enlargement of aerosol particles 2) with no influence on spatial distribution. The 1HP was proven in a plastic box model. PIPAC and ePIPAC were with 60ml of 0.9% NaCl and 10min exposure (EX). ePIPAC comprises two groups: 1^st^ - electrostatic charge before and 2^nd^ - after aerosolisation. Mean aerosol diameter (MAD) and overtime transmission (OT) were measured by Malvern Spraytec®. The 2HP was proven in the flat (2D) and conic folded (3D) blotting paper. 20ml of blue ink were aerosolised during PIPAC and ePIPAC (see group 1). Photos were taken and relative integrated density (RID) was calculated by ImageJ® and compared between 3 zones (see figure 1).

**Results**: In PIPAC, the MAD was 25.691im during aerosolisation, increasing to 99.531im during 10min EX. In ePIPAC 1^st^ group, 26.471im, increasing to 28.361im during EX; in 2^nd^ group, 26.561im, increasing to 26.761im during EX. OT was below the initial ground for 506sec after PIPAC, 23sec after ePIPAC group one and 16sec after ePIPAC group two. RID in 2D PIPAC was in zone one 20%, zone two 40% and zone three 40%; in 2D ePIPAC 30%, 42% and 28%. RID in 3D PIPAC between zones was 23%, 38% and 40%; in ePIPAC 20%, 47% and 33%. During aerosolisation was no meaningful difference in MAD between PIPAC and ePIPAC. During the EX, MAD increases drastically by 3.9 times in PIPAC, but not in ePIPAC 1.1 and 1.0. Aerosol floating 22-32 times longer after PIPAC vs ePIPAC. Ink distribution between zones was the same in 2D and 3D PIPAC 2:4:4, but not in 2D and 3D ePIPAC 3:4:3 and 2:5:3.

**Conclusion**: Electrostatic precipitation prevents the enlargement of aerosol particles during PIPAC and can significantly shorten exposure time Ink distribution was inhomogeneous after PIPAC and ePIPAC.

## ISSPP 2022 POSTER ABSTRACT 25.

### QUANTIFYING PERITONEAL DISEASE DURING PRESSURIZED INTRA-PERITONEAL AEROSOLIZED CHEMOTHERAPY (PIPAC): A NOVEL COMPUTER-BASED SCORING MODEL

Jolene Wong^1^, Wim Ceelen^1^, Wouter Willaert^1-2^

^1^Department of GI Surgery, Ghent University Hospital, Belgium; ^2^Department of Human Structure and Repair, Ghent University, Belgium

**Introduction**: Sugarbaker’s Peritoneal Cancer Index (PCI) is one of the most frequently utilized scores amongst peritoneal surface malignancy specialists worldwide. It represents a standardized way of reporting the extent of peritoneal involvement intra-operatively during cytoreductive surgery and hyperthermic intra-peritoneal chemotherapy (CRS and HIPEC). Pressurized Intra-peritoneal Aerosolized Chemotherapy (PIPAC) is a novel mode of intra-peritoneal drug delivery that has been increasingly adopted amongst patients with extensive peritoneal metastases (PM). It boosts improved tissue distribution, penetration, and repeatability via a laparoscopic approach. Currently, response to sequential PIPACs have been quantified using the PCI score. However, this is not ideal as the PCI was developed as a ‘once-off’ assessment score for CRS and cannot quantify smaller PM volume changes following sequential PIPACs. Therefore, we propose a novel digital way of quantifying the volume of PM during PIPAC and aim to determine the feasibility, and reliability of the digitally determined peritoneal metastases surface area (PMSA) scor**e** amongst PM patients.

**Methods**: In this pilot, we review video recordings of patients undergoing sequential electrostatic-PIPAC (e-PIPAC). A novel digital PMSA score was developed by specialist-PIPAC surgeons based on clinical experience (Figure 1). PMSA scores (score range 0 to 100) were tabulated by 2 independent surgeons using *Image J*, and region of interests (ROI) drawn around tumor deposits to determine the proportion of surface area (SA) involvement in each of the 5 key parietal peritoneal areas. Inter-observer scores were compared and correlation with PCI score performed.

**Results**: Fifteen videos of 5 patients undergoing 3 consecutive PIPACs over a 6-months duration were reviewed. 3 patients had hepatobiliary primaries, 1 esophageal and 1 gynecological. Mean PMSA score was 15 out of 100 (SD 12.6) while mean parietal PCI score was 10 out of 27 (SD 3.5). In general, PCI and PMSA scores followed a similar trend especially in patients with low volume disease. Amongst individual PIPAC sessions with stable or only minimal change in PCI score, PMSA score revealed greater variability. There was good inter-observation correlation in PMSA scores tabulation and mean duration of imaging analysis was 15 minutes (range 10 to 20).

**Conclusion**: The novel digital PMSA score developed for the evaluation of PM burden during sequential PIPACs is feasible and reproducible.

Figure 1. Illustration of 5 Key Parietal Peritoneal Areas in PMSA

**Figure:**
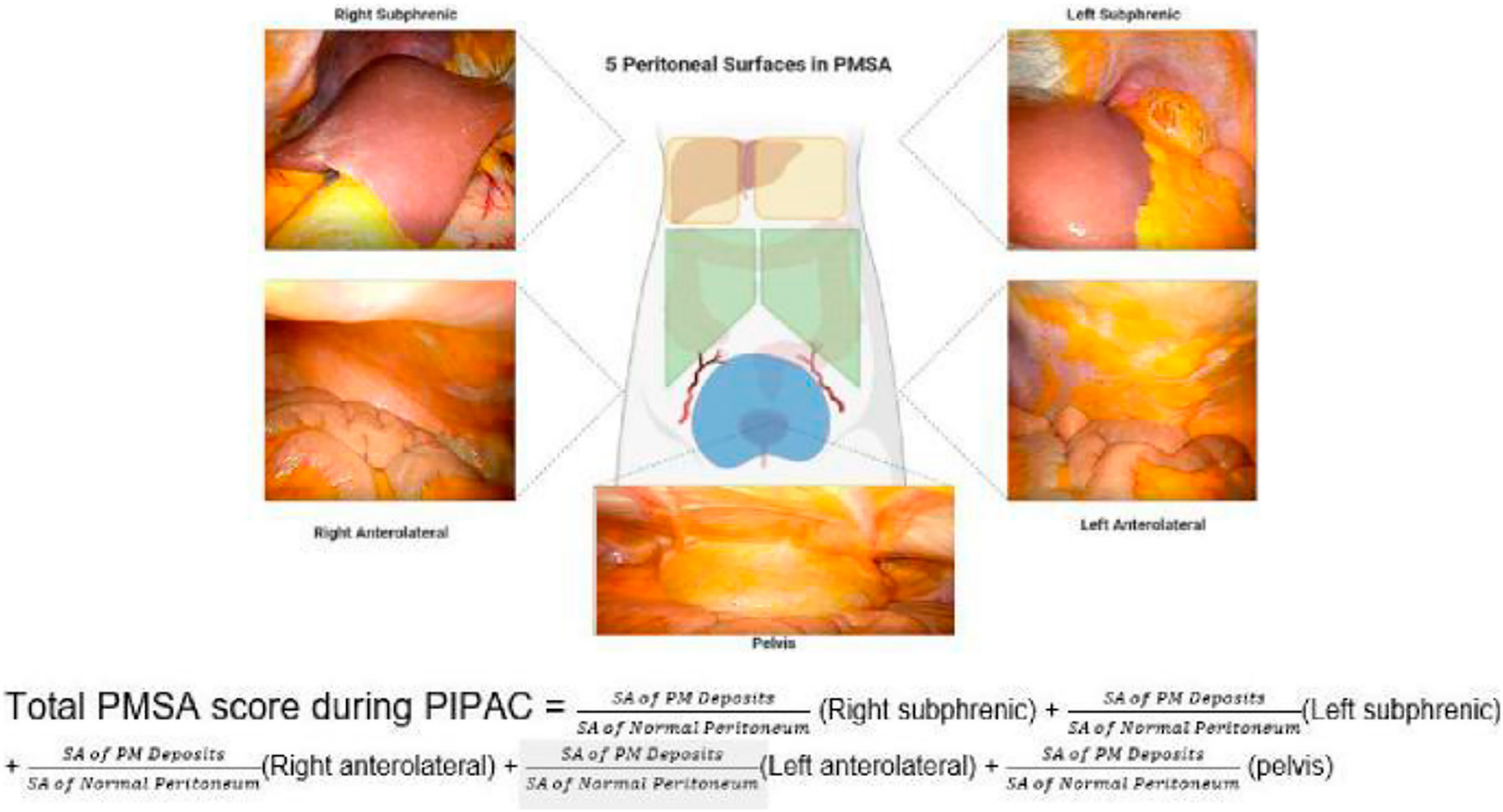


## ISSPP 2022 POSTER ABSTRACT 26.

### UNRAVELING THE IMMUNE TUMOR MICROENVIRONMENT OF COLORECTAL PERITONEAL METASTASES

Sam Ernst ^1,2,3^; Jesse Demuytere^1,3^, Els Lebegge^4,5^; Jan Brughmans^4,5^; Damya Laoui^4,5^; Jo Van Ginderachter ^4,5^; Olivier De Wever^2,3^; Wim Ceelen^1,3^

^1^Laboratory of Experimental Surgery, Department of Human Structure and Repair, Ghent University, Ghent, Belgium; ^2^Laboratory of Experimental Cancer Research (LECR), Department of Human Structure and Repair, Ghent University, Ghent, Belgium; ^3^Cancer Research Institute Ghent (CRIG), Ghent University, Ghent, Belgium; ^4^Laboratory of Cellular and Molecular Immunology, Vrije Universiteit Brussel, Brussels, Belgium; ^4^Myeloid Cell Immunology Laboratory, VIB Center for Inflammation Research, Brussels, Belgium

**Introduction**: Current therapies for colorectal cancer (CRC) peritoneal metastasis (PM) lack sufficient efficacy, hence the need for new therapeutic strategies. However, very little is known on the tumor microenvironment (TME) composition of CRC PM lesions. Specifically, the immune contexture of colorectal PM has not been characterized in detail.

**Methods**: From patients providing informed consent, fresh surgical PM samples were obtained from three anatomical locations: the abdominal wall (AW), the small bowel mesentery, and the greater omentum. Normal ‘*healthy*’ peritoneum was taken from the same patients as control tissue. The resected tumor tissue was analyzed using flow cytometry and cytokine and chemokine analysis of the secretome.

**Results**: In all three PM locations and in the normal peritoneal samples, a prominent population of immune cells could be observed, representing all major immune cell types (granulocytes, lymphocytes, and myeloid cells). Despite considerable inter- and intrapatient heterogeneity, some trends could be observed. In general, the AW metastases comprised fewer immune cells compared to lesions sampled from the other locations. Granulocytes were well represented in the tumor nodules, with neutrophils more common than eosinophils. Macrophages were also commonly present in the TME, whereas monocytes were not. Within the adaptive immune system, a significant number of T cells could be observed, with the majority consisting of cytotoxic T cells, whereas only few B cells were present. Secretome analysis showed a strong inflammatory TME and a chemokine secretome conductive to recruit immune cells.

**Conclusion**: The TME of colorectal PM is characterized by a prominent immune cell population, and by a secretome that is capable of recruiting immune cells. Further functional analysis is ongoing.

## ISSPP 2022 POSTER ABSTRACT 27.

### PERITONEAL REGRESSION IN PATIENTS HAVING PRESSURIZED INTRAPERITONEAL CHEMOTHERAPY FOR PERITONEAL METASTASES

Hugo Teixeira Farinha^1^, Melina Deban^2^, Mohammad Alyami^3^, Naoual Bakrin^4^, Manuela Robella^5^, Olivia Sgarbura^6^, Abdelkader Taibi^7^ and Martin Hübner^1^

^1^Department of Visceral Surgery, Lausanne University Hospital (CHUV), University of Lausanne (UNIL), Lausanne, Switzerland; ^2^Section of Surgical Oncology, Department of Surgery, University of Calgary, Calgary, Alberta, Canada.; ^3^Department of General Surgery and Surgical Oncology, Oncology Center, King Khalid Hospital, Najran, Saudi Arabia; ^4^Department of General Surgery and Surgical Oncology, Centre Hospitalier Lyon-Sud, Hospices Civils de Lyon, Pierre-Bénite, France, EMR 3738 Lyon Sud Charles Mérieux Faculty, Claude Bernard University Lyon 1, France; ^5^Unit of Surgical Oncology, Candiolo Cancer Institute, FPO-IRCCS, 10060 Candiolo, Italy; ^6^Department of Surgical Oncology, Cancer Institute Montpellier, Montpellier, France; ^7^Digestive Surgery Department, Dupuytren Limoges University Hospital, Limoges, France.

**Introduction**: The Peritoneal Regression Grading Score (PRGS) has been elaborated to

assess histological response of peritoneal metastases (PM) to chemotherapy. The aim of this study was to study PRGS before and after repeated intraperitoneal aerosolized

chemotherapy (PIPAC) and to correlate histological response with survival by disease entity.

**Methods**: This retrospective cohort study included consecutive patients from 6 expert

centers having at least 3 PIPACs. Patients with missing information for PRGS at PIPAC1 and 3 (ΔPRGS) and overall survival from date of PIPAC1 were excluded. A sensitivity

analysis of PRGS was performed regarding histology, bimodal treatment (PIPAC concurrent with intravenous chemotherapy), PCI and cytology.

**Results**: A total of 223 patients were identified. 48% (n=108) were male and median (IQR) age was 59.9 (51-69) years. Histological regression under PIPAC was observed in 49% (n=26) of patients with colorectal PM, 44% (n=7) with appendix, 44% (n=38) with gastric, 49% (n=17) with ovarian, 45% (n=9) with mesothelioma and 67% (n=8) with hepatobiliary. Bimodal treatment was associated with increased ΔPRGS only for the colorectal PM subgroup (p=0.0095). No correlation was seen between ΔPRGS and entity (p=0.84), PCI (p=0.67), bimodal treatment (p=0.13) or cytology (p=0.06). No correlation was observed between histological regression and survival for either entity (p=0.89).

**Conclusion:** Approximately half of patients with PM treated with 3 PIPACs have a histological response to chemotherapy. Change in PRGS was not correlated with survival.

## ISSPP 2022 POSTER ABSTRACT 28.

### PROPHYLACTIC HYPERTHERMIC INTRAPERITONEAL CHEMOTHERAPY (HIPEC) FOR CLINICAL T4 GASTRIC CANCER MIGHT IMPROVE OVERALL AND DISEASE-FREE SURVIVAL: SINGLE CENTER EXPERIENCE

Sheng-En Chou^1^, Shih-min Yin^1^, Yueh-Wei Liu^1^, Yu-Yin Liu^1^

^1^Department of General Surgery, Kaohsiung Chang Gung Memorial Hospital and Chang Gung University College of Medicine, Kaohsiung, Taiwan

**Introduction**: Patients with clinical T4 gastric cancers usually have low overall survival (OS) and high-risk for recurrence. The data of HIPEC as prophylactic treatment in clinical T4 gastric cancer is still limited

**Methods**: From 2018-2022, we retrospective review gastric cancer patients underwent curative resection and HIPEC as prophylactic treatment. The inclusion criteria for prophylactic HIPEC are 1) clinical T4 stage patients, 2) patients with cytological evidence of micro-metastasis, and 3) age <80-year-old. Postoperatively, all patients were followed for at least 12 months.

**Results:** Total 26 gastric cancer patients underwent HIPEC were carefully reviewed. There are 11 patients with clinical T4 gastric cancer receive prophylactic HIPEC after curative resection. The prophylactic HIPEC regimen was cisplatin 150 mg (60 min, 42 ± 1C), and three patients had additional mitomycin-C 30mg. Radical subtotal gastrectomy was performed in 10 patients and only 1 total gastrectomy was performed. No in-hospital mortality but one anastomostic leakage was reported. The 1-year and 3-year OS is 100% and 72.9%, and the 1-year and 3-year DFS is 90.9% and 77.9%. All patient receive further adjuvant chemotherapy or immunotherapy postoperatively.

**Conclusions:** For patients with clinical T4 gastric cancer, prophylactic HIPEC plus radical gastrectomy seem a promising option to prevent peritoneal recurrence and improve OS and DFS.

## ISSPP 2022 POSTER ABSTRACT 29.

### SURVIVAL OUTCOMES AND MAJOR MORBIDITY IS NOT ASSOCIATED WITH ADVANCED AGE IN PATIENTS UNDERGOING CYTOREDUCTIVE SURGERY AND HYPERTHERMIC INTRAPERITONEAL CHEMOTHERAPY: A SINGLE CENTRE EXPERIENCE

Raymond Hayler^1,2^, Ernest Cheng MD^1,2^, Raphael Shamavonian MBBS^1^, Jasmine Mui MD^1,2^, Josh Karpes MBBS1,2, ^,2^, Ruwanthi Wijayawardana^1^, Shoma Barat^1^, Nima Ahmadi MBBS FRACS^1^, David L. Morris MB, ChB, FRCS, MD, PhD^1,2^

^1^Peritonectomy and Liver Cancer Unit, Department of Surgery, St George Hospital, Kogarah, NSW, Australia; 2St George Hospital Clinical School, University of New South Wales, Kogarah, NSW Australiaen

**Introduction:** Peritoneal carcinomatosis is associated with poor prognosis without invasive and aggressive surgical management in the form of Cytoreductive surgery (CRS) and Hyperthermic intraperitoneal chemotherapy (HIPEC). Advanced age is often associated with increased risk of morbidity and mortality for major oncological intra-abdominal surgeries. This study investigates the short- and long-term outcomes of patients over the age of 70 undergoing CRS/HIPEC.

**Methods:** A retrospective cohort analysis was performed on a single institution database of CRS/HIPEC patients from January 1996 to March 2022. Patients were categorised by age ^3^ 70 or < 70. The primary outcome was long term overall survival (OS). Secondary outcomes included morbidity, mortality, hospital length of stay, intensive care (ICU) length of stay and further treatment with early postoperative intraperitoneal chemotherapy (EPIC).

**Results:** A total of 1129 patients were identified of which 134 were aged 70 and over and 935 were under 70. Median OS was 34.5 months for the older group and 37.7 months for the younger group (p = 0.961). Of the patients aged 70 and over, 38.1% of them experienced a major complication compared to 32.9% in the younger cohort (p = 0.137). Advanced age was associated with higher mortality (4.48 vs 1.11, p =0.010), longer ICU stay (7.72 vs 4.26 days, p < 0.001), longer hospitalization (37.2 vs 26.13 days, p<0.001) and were less likely to receive EPIC (23.9% vs 32.7%, p = 0.040).

**Conclusion:** In patients with peritoneal carcinomatosis undergoing CRS/HIPEC, age 70 and over does not impact on overall survival or major morbidity. Therefore, age alone should not be used as a limiting factor in selecting patients, however, a careful multi-disciplinary approach should be taken.

## ISSPP 2022 POSTER ABSTRACT 30. PAP.2022.0161

### BIDIRECTIONAL APPROACH WITH PIPAC AND SYSTEMIC CHEMOTHERAPY FOR PATIENTS WITH SYNCHRONOUS GASTRIC CANCER PERITONEAL METASTASES (GCPM): COULD BE THE GATE TO CONVERSION SURGERY?

Francesco Casella^1^, Maria Bencivenga^1^, Giorgio Brancato^1^, Michele Sacco^1^, Lorena Torroni^2^, Cecilia Ridolfi^1^, Carmelo Puccio^1^, Giuseppe Verlato^2^, and Giovanni de Manzoni^1^

^1^Upper G.I. Surgery Division, University of Verona, Piazzale Aristide Stefani 1, 37126, Verona, Italy; ^2^Department of Diagnostics and Public Health University of Verona, Strada le Grazie, 8, 37134, Verona, Italy

**Introduction**: The aim of this study was to evaluate the efficacy of PIPAC in combination with systemic chemotherapy as bidirectional approach for gastric cancer patients with peritoneal metastases.

**Methods**: A retrospective analysis of a prospective PIPAC database was queried for patients with gastric cancer peritoneal metastasis who underwent PIPAC with cisplatin and doxorubicin and systemic therapy between June 2019 and May 2022 at the Upper Gastrointestinal Surgery of Verona. Surgical and oncological short-term outcomes are reported. The Peritoneal Regression Grading Score (PRGS) was used to assess the pathologic response of patients who underwent more than one PIPAC.

**Results**: Sixty-one PIPAC in 32 patients were performed during systemic chemotherapy. Nine patients underwent only 1 PIPAC, seventeen patients 2 PIPAC and six patients 3 PIPAC. Twenty two patients received FOLFOX scheme, five patients FLOT, two patients TCF, two patients received Ramucirumab-paclitaxel and one patient XELOX before first PIPAC. Six patients (18,7%) underwent total gastrectomy with D2 lymphadenectomy and HIPEC was added in 4 cases. R0 was achieved in 5 cases, the remaining had R1 resection. Major complications (CD ≥3a) was recorded only in one procedure (1,6%). The median overall survival was 17,9 months.

**Conclusions**: In our experience bidirectional therapy with PIPAC and systemic chemotherapy is a safe and effective therapeutical strategy in treatment of gastric cancer patients with synchronous peritoneal metastases. In selected patient this approach could be considered the gate to conversion surgery.

## ISSPP 2022 POSTER ABSTRACT 31. PAP.2022.0168

### PRESSURIZED INTRAPERITONEAL AEROSOL CHEMOTHERAPY (PIPAC) FOR PERITONEAL MALIGNANCIES: 3 YEARS’ EXPERIENCE FROM AN ITALIAN DEDICATED CENTER

Francesco Casella^1^, Giorgio Brancato^1^, Maria Bencivenga^1^, Michele Sacco^1^, Lorena Torroni^2^, Cecilia Ridolfi^1^, Carmelo Puccio^1^, Giuseppe Verlato^2^, and Giovanni de Manzoni^1^

^**1**^Upper G.I. Surgery Division, University of Verona, Piazzale Aristide Stefani 1, 37126, Verona, Italy; ^2^Department of Diagnostics and Public Health University of Verona, Strada le Grazie, 8, 37134, Verona, Italy

**Introduction**: Peritoneal malignancies include primary as well as metastatic tumors of the peritoneum. Irrespective of the origin, peritoneal metastases represent an advanced stage of disease and are associated with poor outcomes. The minimally invasive approach of Pressurized IntraPeritoneal Aerosol Chemotherapy (PIPAC) allows the repeated applications of chemotherapeutic agents directly into peritoneal cavity and at the same time an objective assessment of tumor response thanks to the possibility to obtain histological samples. This study aimed to investigate the experience of a single dedicated center.

**Methods**: This retrospective cohort study included consecutive patients treated with PIPAC at a single center from May 2019 to May 2022. The toxicity of each procedure was assessed using the Common Terminology Criteria for Adverse Events (CTCAE). Complications were reported according to the Clavien-Dindo classification. Quality of life (QoL) was assessed using EORTC-QLQ-C30, and the peritoneal regression grading score (PRGS) was used to grade histologic responses. Oncological and clinical outcomes were analyzed.

**Results**: Overall, 80 patients received 131 PIPACs with cisplatin/doxorubicin (7.5mg/m2 + 1.5 mg/m2 n = 31; 10.5mg/m2 + 2.1 mg/m2 n = 94) or oxaliplatin 92,5 mg/m2 (n = 6) for gastric (n = 60), colorectal (n = 5), breast (n = 5), HBP (n = 3), MPM (n = 2), ovarian (n = 2), PMP (n = 2) and small bowel (n = 1) primary cancers. Systemic chemotherapy was used in all patients, 40 (50%) of these in bidirectional approach. Median PCI was 24 (2-39) and fifty-two patients had ascites at the first PIPAC administration, with a median volume of 1400 ml (min-max: 50-9000 ml). The complete (PRGS1) and major (PRGS2) histologic responses at the second PIPAC were 14,2% and 46.4% respectively, while after the third PIPAC they were 25% and 37,5%. In 13 procedures bilateral adnexectomy for Krukenberg lesions was performed before PIPAC in the same operation. After a bidirectional approach, 8 patients (10%) underwent to CRS (7 patients total gastrectomy with D2 lymphadenectomy and 1 patient small bowel resection), HIPEC was added in 5 cases. Median hospital stay was 1,7 days and the in-hospital mortality rate was 0%. The overall and major CTCAE toxicity rates were respectively 14.6% and 8.9%. The postoperative complications rate according to Clavien classification were 11,5%, major complications (CD ≥3a) occurred only 1 case. Median overall survival from diagnosis was 15,1 months (table 1).

**Conclusion**: In our experience the use of PIPAC is a safe and effective therapeutical strategy in treatment of patients with peritoneal malignancy. It is essential to stratify the patients for PIPAC treatment in palliative, bidirectional or neoadjuvant settings.

## ISSPP 2022 POSTER ABSTRACT 32. PAP.2022.0173

### THE EFFECTS OF MALIGNANT ASCITES AND AN ACIDIC ENVIRONMENT ON TUMOR PHENOTYPE

QianLu Yang^1^, Frank-Jürgen Weinreich^1^, Giorgi Nadiradze^1,2^, Rami Archid^1,2^, Christoph Trautwein ^3^, Stefan Kommoss^4^, Alfred Königsrainer^1,2^, Marc A. Reymond^1,2^.

^1^National Center for Pleura and Peritoneum, NCT South-West Germany, Tübingen; ^2^Dept. of General and Transplant Surgery, University Hospital, Tübingen, Germany; ^3^Werner Siemens Imaging Center, University of Tübingen, Germany; ^4^Women’s Hospital, University of Tübingen, Germany

**Background**: In cancer patients, peritoneal metastasis with malignant ascites contributes to poor prognosis. Cancer cells that shed into the peritoneal cavity need to resist to anoikis and escape from immune elimination. For this purpose, they modify their metabolism to adapt to the hostile environment. There is little data on how ascites influence on tumor phenotype.

**Methods**: Intraoperative measure physical-chemical parameters of malignant ascites of gastric and ovarian cancer vs. lavage of control patients. The pH of the extracellular fluid was adjusted to 6.0, 6.5, 7.0, 7.5, and 10% malignant ascites was added to each gradient. Human gastric (MKN45) and ovarian (OAW42) cell lines were observed for metabolic assays (MTT), cell adhesion assays (adhesion assay), scratch assays for cell migration (Cell Zen Owl), and apoptosis assays (flow cytometry).

**Results**: pH was significantly higher in cancer patients (7.68 ± 0.33) vs. controls (6.87 ± 0.02), p< 0.001. Spearman analysis showed a positive correlation between pH and stage of patients (p<0.05). Metabolic activity in vitro increased with pH in both cell lines (ANOVA, MKN45: p < 0.001, OAW42: p < 0.001), as did cell adhension (MKN45: p < 0.001, OAW42: p < 0.001) and migration (MKN45: p < 0.001, OAW42: p < 0.001). An acidic pH correlated with a higher apoptotic rate (MKN45: p < 0.05, OAW42: p < 0.05). However, malignant ascites helped the gastric and ovarian cancer cells to resist the inhibitory effect of pH alteration on various functions (p<0.05).

**Conclusion**: Intraperitoneal pH is more alkaline in cancer patients vs. controls, and pH was positive correlation with stage. In vitro, cell survival, adhesion, migration, and metabolic activity are impaired in the presence of a lower extracellular pH, but ascites can help cancer cell resist the inhibitory effect of pH.

## ISSPP 2022 POSTER ABSTRACT 33. PAP.2022.0175

### PRESSURIZED INTRAPERITONEAL AEROSOL CHEMOTHERAPY (PIPAC) IN GASTRIC AND COLORECTAL CANCER PATIENTS WITH PERITONEAL METASTASIS – A PALLIATIVE TREATMENT STRATEGY WITH NEOADJUVANT POTENTIAL

Daniel Chourio Barboza^1^, Jan-Philipp Ramspott^2^, Patrycja Slepecka^3^, Tobias Nowacki, Tobias^4^, Andreas Pascher^1^, Judith Sporn^1^

^1^University of Münster Faculty of Medicine, Department of Surgery; ^2^University of Münster Faculty of Medicine, Department of Surgery; ^3^ University of Münster Faculty of Medicine, Department of Surgery; ^4^ University of Münster Faculty of Medicine, Department of Gastroenterology; ^5^University of Münster Faculty of Medicine, Department of Surgery.

**Introduction**: The peritoneum is a frequent site of disease progress or tumor recurrence in gastrointestinal malignancies. Most patients with peritoneal metastasis (PM) are not eligible for surgical resection. Pressurized intraperitoneal aerosol chemotherapy (PIPAC), has been introduced as a novel technique for the treatment of cancer patients with PM. Current evidence demonstrates its safety and efficacy with regard to symptomatic control, pathological and radiological regression. The goal of this study is to present our experience with PIPAC as part of a multidisciplinary approach in treating gastric and colorectal cancer patients with PM.

**Methods**: We conducted a retrospective analysis, which included all patients who underwent PIPAC as part of a multidisciplinary approach for the treatment of gastric or colorectal cancer with PM since April 2020. PIPAC was performed at a pressure of 12 mmHg over 30 minutes. For gastric cancer patients, we used doxorubicin and cisplatin at a concentration of 2.1 and 10.5mg/m^2^ BSA. For colorectal cancer patients, we used oxaliplatin at either 92 or 120mg/m^2^ BSA.

**Results**: We performed a total of 53 procedures in 32 patients, 12 with gastric, 20 with colorectal cancer and PM. The median PCI at the time of first PIPAC was 17. 17 (53.1%) patients underwent one PIPAC procedure, and 15 (46.9%) underwent two or more. The median age was 60 years (range 25-82). The overall morbidity wasC 21.8% (7 patients), which included 4 patients with grade 1-2 complications (12.5%), 3 patients with a grade 3-4 complication (9.3%), and no patient with grade 5 complications (0% mortality rate). Of the 32 patients who underwent PIPAC therapy as an -initially- palliative treatment approach, 7 (21.8%) could eventually be treated with CRS and HIPEC with curative intent.

**Conclusion**: PIPAC is a safe and effective tool for the treatment of gastric and colorectal cancer patients with PM. In addition to its role in the palliative treatment of PM, PIPAC has the potential to be used as a pseudoneoadjuvant strategy to render patients eligible for CRS and HIPEC with curative intent.

## ISSPP 2022 POSTER ABSTRACT 34. PAP.2022.0177

### PROGNOSTIC FACTORS AND SURGICAL OUTCOMES OF CONVERSION GASTRECTOMY FOR PATIENTS WITH GASTRIC CANCER WITH PERITONEAL METASTASES

Daryl KA Chia FRCS^1^ , Raghav Sundar MRCP^2,3,4^, Guowei Kim FRCS^1^ , Jia Jun Ang MRCS^1^, Jeffrey HY Lum FRCPath^5^ , Min En Nga FRCPath^5^ , Giap Hean Goh FRCPath^5^, Seet Ju Ee FRCPath^5^, Cheng Ean Chee MRCP^2^ , Hon Lyn Tan MRCP^2^, Jingshan Ho MRCP^2^, Natalie YL Ngoi MRCP^2^, Matilda XW Lee MRCP^2^, Vaishnavi Muthu MRCP^2^, Gloria HJ Chan MRCP^2^, Angela SL Pang MRCP^2^, Yvonne LE Ang MRCP^2^, Joan RE Choo MRCP^2^, Joline SJ Lim MRCP^2^, Jun Liang Teh FRCS^6^, Aung Lwin FRCS^6^, Yuen Soon FRCS^6^, Asim Shabbir FRCS^1,3,7^, Jimmy BY So MPH, FRCS^1,3,7^, Wei Peng Yong MRCP2, ^8^

^1^Department of Surgery, University Surgical Cluster, National University Health System, Singapore; ^2^Department of Haematology-Oncology, National University Cancer Institute National University Health System, Singapore; ^3^Yong Loo Lin School of Medicine, National University of Singapore, Singapore; ^4^The N.1 Institute for Health, National University of Singapore, Singapore; ^5^Department of Pathology, National University Hospital, National University Health System, Singapore; ^6^Department of General Surgery, Ng Teng Fong General Hospital’7Division of Surgical Oncology, National University Cancer Institute, National University Health System, Singapore; ^7^Cancer Science Institute of Singapore, National University of Singapore, Singapore

**Introduction**: Conversion gastrectomy is increasingly being considered for gastric cancer peritoneal metastases (GCPM) patients who have good response to intraperitoneal paclitaxel (IP-PTX) with systemic therapy. However, the outcomes of surgery are unclear. Our study aimed to evaluate surgical outcomes and prognostic factors for conversion surgery.

**Methods**: Patients with GCPM were recruited for a prospective phase II trial and received IP-PTX with oral capecitabine and intravenous oxaliplatin (XELOX) in 21-day cycles. Those with good response to chemotherapy, had negative peritoneal fluid cytology with no extraperitoneal metastases and no carcinomatosis peritonei on re-look diagnostic laparoscopy underwent conversion gastrectomy. Primary outcome was overall survival (OS) and secondary endpoint were morbidity and especially those with Clavien-Dindo IIIb & Above.

**Results**: Of 64 patients with synchronous GCPM, 20 (31.3%) underwent conversion gastrectomy. Median operative time was 316 minutes (IQR 279-368) and median length of stay was 9 days (IQR 7-15). Distal gastrectomy was performed in 45% (9/20) while 55% (11/20) underwent total gastrectomy, with 85% (17/20) performed as open procedure. No combined organ resection or 30-day mortality was noted. Median lymph node harvest was 37 (IQR 23-44) and R0 resection margin was achieved in 65% (13/20) of patients but did not significantly influence median OS (R0 vs. R1-2, median OS; 29.5 vs. 20.7 months, p=0.442). Overall morbidity was 35% (7/20) & major morbidity reported in 10% (2/20) of patients who underwent re-operation for duodenal stump leak and bleeding. The overall 12-month OS was 85% and 24-months OS was 50%. Patients with poorer response to pre-operative therapy (tumour response grading [TRG] <3, p=0.082) and presence of LVI (p=0.057) were found to be associated with OS <24 months although significance was not reached. On survival analysis, median OS for patients with good response to pre-operative treatment (TRG <3) and those who did not (TRG=3) were 28.1 months and 16.0 months respectively (TRG<3, HR 0.085, 95% CI 0.016-0.44).

**Conclusions**: Conversion gastrectomy is a safe and feasible option for select GCPM patients following IP-PTX with systemic treatment. Response to pre-operative treatment was a significant predictor in overall survival after conversion surgery.

## ISSPP 2022 POSTER ABSTRACT 35.

### IMAGING GASTRIC CANCER METASTASIS PROGRESSION IN AN ORGANOTYPIC, THREE-DIMENSIONAL FUNCTIONAL MODEL OF THE HUMAN PERITONEUM

Arianna Castagna^1,4^, Frank-Jürgen Weinreich^1,4^, Hannah Heejung Lee ^1,4,^ Birgit Schittek ^3^, Alfred Königsrainer^1,4^, Marc André Reymond^1,4^, Wiebke Solass^3,4,5^

^1^Dept. of General and Transplant Surgery - University Hospital, Tübingen, Germany ^2^Dept. of Dermatology - University Hospital, Tübingen, Germany ^3^Institute of Pathology and Neuropathology - University Hospital, Tübingen, Germany ^4^National Center for Pleura and Peritoneum, University Hospital, Tübingen, Germany ^5^Institute of Pathology University Bern, Switzerland

**Background**: Peritoneal metastasis (PM) of gastric cancer (GC) has a poor prognosis. Better therapies need a better understanding of the pathogenesis of PM, which in turn requires adequate functional models.

**Methods**: We build a three-dimensional model of the human peritoneum with peritoneal mesothelial cells (PMCs), fibroblasts, endothelial cells and collagen. Then performed imaging with optical microscopy, immunohistochemistry, scanning (SEM), and transmission (TEM). Moreover, we studied the functional capability analysing adhesion, invasion and growth essays.

**Results**: The 3D reconstruction showed a morphology largely comparable with the normal human peritoneum, including the formation of a basal membrane, apical microvillosities and intercellular junctions (TEM). After seeding GC cells (MKN45) onto the peritoneal surface, we observed the adhesion, migration, and invasion (MMPs) of GC cells (MKN45), leading to PM development. Matching images obtained from surgical samples with the model showed a strong analogy.

**Conclusion**: This powerful model will allow morphological and functional imaging of the effect of physical, chemical, and pharmacological interventions on the progression of PM in GC.

## ISSPP 2022 POSTER ABSTRACT 36.

### CONTEMPORARY NATIONAL PRACTICE PATTERNS AND SURVIVAL IN MALIGNANT PERITONEAL MESOTHELIOMA

Lucia Calthorpe^1^, Fernanda Romero-Hernandez^1^, Megan Casey^1,^ ^2,^ ^3,^ Amir Ganjouei^1^, Jaeyun Wang^1^, Alex Kim^4^, Carlos Corvera^1^, Adnan Alseidi^1^,

^1^University of California San Francisco, Department of Surgery; ^2^University of California San Francisco, School of Medicine Nunez, Miguel; ^3^University of California San Francisco, School of Medicine Conroy, Patricia; ^4^The Ohio State University, Department of Surgery

**Introduction**: Malignant peritoneal mesothelioma (MPM) is a rare malignancy with a historically poor prognosis. Cytoreductive surgery (CRS) with hyperthermic intraperitoneal chemotherapy (HIPEC) has emerged as an effective therapy for patients with peritoneal malignancies. A contemporary analysis of trends in the management and survival of MPM is warranted.

**Methods**: Patients with MPM were identified from the National Cancer Database (2004-2018). Patients were categorized by treatment (CRS-HIPEC, CRS-Chemotherapy, CRS only, Chemotherapy only, and no treatment). Joinpoint regression was employed to compute the annual percent change (APC) in treatment over time. Multivariable Cox proportional hazards models were used to analyze factors associated with survival.

**Results**: Of 2,683 patients with MPM, 19.1% underwent CRS-HIPEC, 20.9% CRS-Chemotherapy, 29.7% Chemotherapy only, 9.2% CRS only, and 21.1% no treatment. Joinpoint regression showed a significant increase in the proportion of patients undergoing CRS-HIPEC over time (Annual Percent Change (APC) 3.21, p=0.01), and a concurrent decrease in the proportion of patients who received no treatment (APC -2.21, p=0.02). Median overall survival was 19.5 months. Factors independently associated with survival included CRS-HIPEC, CRS, histology, sex, age, race, Charlson Comorbidity Index, insurance, and hospital type. Although there was a strong association between year of diagnosis and survival on univariate analysis (2016-18 HR 0.67, p<0.001), this association was attenuated after adjustment for treatment.

**Conclusions**: CRS-HIPEC is increasingly employed as a treatment for MPM. There has been a decrease in patients receiving no treatment with a concomitant increase in overall survival. These findings suggest that patients with MPM may be receiving more appropriate therapy, leading to improvements in survival over time.

## ISSPP 2022 POSTER ABSTRACT 37.

### IN VITRO TOXICITY AFTER PRESSURIZED INTRAPERITONEAL AEROSOL CHEMOTHERAPY WITH AND WITHOUT ELECTROSTATIC PRECIPITATION VS HYPERTHERMIC INTRAPERITONEAL CHEMOTHERAPY

Iaroslav Sautkin^1^, Alfred Koenigsrainer ^1^, Marc Reymond^2^

^1^ University Hospital Tuebingen, Department of General, Visceral and Transplant Surgery; ^2^University Hospital Tuebingen, Dept. of Surgery and Transplantation;

**Introduction**: Cell toxicity is an important parameter showing the effectiveness of antitumor therapy. There is no data about cell toxicity after ePIPAC and no comparison between PIPAC vs ePIPAC vs HIPEC. The obtained data can help optimize pharmacological and environmental aspects of treatment and improve clinical outcomes.

**Methods**: Null hypothesis: there is no difference in cell toxicity after PIPAC vs ePIPAC vs HIPEC. Cell toxicity was observed in cell growth inhibition study and MTT viability assay. The electrostatic cell culture model was established for ePIPAC. Immortal normal human fibroblasts were plated. In PIPAC and ePIPAC the concentration of doxorubicin was 2.49x10^-5^mol/L and cisplatin 2.25x10^-4^mol/L. In HIPEC, the doxorubicin concentration was 1.38x10^-5^mol/L and cisplatin 1.17x10^-4^mol/L. All controls with 0.9% NaCl. Biological replication with 3 passages, technical with 6-well plate. Exposure time after PIPAC and ePIPAC was 30min. Electrostatic charge in ePIPAC was during the exposure time. HIPEC was at 42°C for 60min. Cell count was on each 3^rd^ day and MTT assay 48h after the experiment.

**Results**: Cell count on 3d after PIPAC vs ePIPAC vs HIPEC: 37.51±29.42% vs 40.88±28.9% vs 35.48±14.48% (p>0.05); 9d: 0.66±1.9% vs 0% vs 24.92±23.74% (PIPAC vs ePIPAC p>0.05; PIPAC vs HIPEC and ePIPAC vs HIPEC <0.001); 12d: 0% vs 0% vs 0%. Controls on 3d: 78.46±39.48% vs 62.60±45.0% vs 99.06±37.48% (ePIPAC vs HIPEC p<0.05); 15d: 99.29±67.30% vs 47.28±32.01% vs 146.42±95.88% (ePIPAC p<0.05). Signs of cell membrane blebbing were in all test groups on day 3 after treatment. Cell viability: 56.51±14.98% vs 51.82±13.83% vs 58.42±12.17% (ePIPAC vs HIPEC p=0.003). Controls: 75.60±29.93% vs 61.15±16.87% vs 70.27±14.71% (ePIPAC vs HIPEC p<0.001; ePIPAC vs PIPAC p<0.001).

Complete cell growth inhibition was on day 9 after ePIPAC and day 12 after PIPAC and HIPEC. In control, a significant cell growth inhibition was after ePIPAC. Cell viability was the lowest after ePIPAC, but significantly only vs HIPEC. In control, cell viability was significantly lower after ePIPAC.

**Conclusion**: The electrostatic precipitation increases the cell toxicity of aerosol chemotherapy. ePIPAC demonstrated the highest cell toxicity. PIPAC and HIPEC showed no significant difference in cell viability.

## ISSPP 2022 POSTER ABSTRACT 38.

### APPENDICEAL ADENOCARCINOMA, DIAGNOSED AFTER ACUTE PERFORATED APPENDICITIS: POTENTIAL CONTRIBUTION OF HIPEC

Elsa Leiritz ^1^, Jérémy Rezai ^2^, Mathilde Wagner ^3^, Armelle Bardier ^4^, Marc Pocard ^1,5^

^1^ University Hospital Pitié Salpêtrière, Surgical Digestive Department; ^2^University Hospital Pitié Salpêtrière, Hepato Gastroenterology Department; ^3^University Hospital Pitié Salpêtrière, Radiology Department; ^5^University Hospital Pitié Salpêtrière, Pathology Department; ^5^Paris 7 University, Surgical unit ; INSERM, U965 CART unit

**Introduction**: Treatment of peritoneal metastasis from appendicular adenocarcinoma consist in cytoreduction surgery (CRS) and HIPEC (chemotherapy hyperthermia intraperitoneal). In case of acute appendicular syndrome (SAA), adenocarcinoma is often discovered on a second step, and the tumor is likely to be perforated. In that case, there is no treatment recommendation, but peritoneal metastasis is concern. We propose a right colectomy, with CRS and HIPEC.

**Methods**: Consecutive 22 patients were addressed for discovery of appendiceal adenocarcinoma. The emergency surgery was performed at the hospital of nearest proximity. We evaluated the therapeutic algorithms, per operative decision, survival and recurrent rate.

**Results**: Four patients diagnosed as synchronous appendicular peritoneal metastasis, had CRS and HIPEC. 2 are in remission (50%, median follow up time 26 months). 18 patients with diagnosis of adenocarcinoma on anatomopathological sample, without peritoneal metastasis during appendectomy and at radiological exams were addressed. 2 patients were recused of CRS. Among the 16 patients operated for CRS and HIPEC : 56% (n=9) had PCI 0, 13% (n=) PCI 1-10, 31% (n=) PCI > 10. Postoperative complications at 90 days, are at 12,5% (n=2) grade 3B, 6,25% (n=1) grade 4, no grade 5. For the 9 patients with prophylactic HIPEC, PCI at zero, the recurrence rate at 5 years is 11,1%, OS is 100%. For the 7 patients with peritoneal metastasis the recurrence rate at 5 year is 85,6%, OS is 14,4%.

**Conclusion**: In case of appendectomy in emergency situation for perforated adenocarcinoma, half of the patient had peritoneal metastasis, whatever the surgeon identify. Referring the patient in a peritoneal surgical unit is mandatory. In case of non-identified peritoneal metastasis at CRS, performing a prophylactic HIPEC is associated with 88,9% of peritoneal disease free at 5 years. Other observational large studies are necessary to comfort that strategy.

## ISSPP 2022 POSTER ABSTRACT 39.

### RETROSPECTIVE COHORT ANALYSIS OF THE TREATMENT OF PERITONEAL MESOTHELIOMA

Hannah H Lee^3,4^, Lucia Eberl ^3,4^,Marc A Reymond ^3,4^, Alfred Königsrainer ^3,4,^ Hans Bösmüller ^1^, Wiebke Solass^1,2^

^1^Institute of Pathology and Neuropathology University Hospital Tübingen: Eberhard-Karls-University Tübingen, Germany; ^2^Institute of Pathology, University Bern, Switzerland; ^3^National Center for Pleura and Peritoneum, University of Tübingen, Germany; ^4^Dept. of General and Transplant Surgery, University Hospital Tübingen, Eberhard-Karls-University Tübingen, Germany

**Introduction**: Peritoneal Mesothelioma is a rare and fatal malignancy with poor prognosis and limited treatment options. Current standard of care is cytoreductive surgery and simultaneous intraoperative HIPEC (hyperthermic intraperitoneal chemotherapy). New treatment options like PIPAC are increasingly used. To evaluate the efficacy of these different treatment regimens regarding overall survival (OS) we conducted a retrospective cohort study.

**Material and Methods:** Unsupervised machine learning analysis in a retrospective single-center cohort study of patients with peritoneal mesothelioma who underwent treatment from 01/2011 to 12/2021. Ethical approval was given (232/2022BO2).

**Results**: 33 patients with peritoneal mesothelioma were included in the study, 20 female and 13 male patients. Unsupervised machine learning clustered the cohort in three subgroups (CRS and HIPEC group, PIPAC group, systemic chemotherapy group) and showed group specific characteristics.58% of patients were eligible for CRS and HIPEC, 24% received systemic chemotherapy and 15% PIPAC, and 3% received other treatment options. Median overall survival for the CRS /HIPEC group was 81 month, 33 months in the PIPAC group and 63 month in the chemotherapy group (one long-term survivor). Histological analysis showed 88% epithelioid, 6% a biphasic and 6% papillary growth pattern.

**Conclusion**: The retrospective analysis of the small cohort showed that CRS/HIPEC and systemic chemotherapy can lead to satisfying results in the treatment of peritoneal mesothelioma. Furthermore, PIPAC can prolong survival in patients with extensive and non-resectable peritoneal mesothelioma. However, due to its rarity large volume data is needed to further investigate the importance of each treatment regimen. Our proposition would be the establishment of a multicenter retrospective database with maintained prospectively data collection to clarify the efficacy of different treatment regimes.

## ISSPP 2022 POSTER ABSTRACT 40.

### VALIDATION OF THE PHASE 1 DOSE ESCALATION STUDY OF OXALIPLATIN, CISPLATIN AND DOXORUBICIN APPLIED AS REPEATED PIPAC IN PATIENTS WITH PERITONEAL SURFACE MALIGNANCIES

Manuela Robella^1^, Michele De Simone^1^, Marco Vaira^1^

^1^Candiolo Cancer Institute – FPO, IRCCS

**Introduction**: In a previous phase I study we reported good results in terms of safety using cisplatin (CDDP) and doxorubicin (DXR) as PIPAC at a dose of 30 mg/sm and 6 mg/sm, respectively; oxaliplatin (OXA) was used at an intraperitoneal dose of 135 mg/sm. The dosages achieved were the highest ever used in PIPAC. Some concerns were raised by the international community about the choice of performing only one PIPAC procedure per patient. This is a validation analysis of our dose escalation study with the aim of confirming feasibility and safety of the identified doses applied as repeated PIPAC.

**Methods**: As off label treatment we planned to treat, in order to support the safety of the dosages achieved, a series of 6 patients with CDDP + DXR at a dose of 15 mg/sm and 3 mg/sm (the second dose escalation step of our phase 1 study); in case of no adverse events a further cohort of 6 patients would be treated with repeated CDDP + DXR PIPAC at a dose of 30 mg/sm and 6 mg/sm, respectively. The starting dose of oxaliplatin was 135 mg/sm. Safety was assessed according to Common Terminology Criteria for Adverse Events (CTCAE version 4.03). Only patients submitted to at least 3 PIPAC procedures were included in the analysis.

**Results**: Six patients were treated with CDDP 15 mg/sm + DXR 6 mg/sm; no major side effects were found. Further six patients were submitted to PIPAC with CDDP 30 mg/sm + DXR 6 mg/sm without major postoperative complications. Common adverse events in both cohorts included postoperative abdominal pain and nausea. Regarding PIPAC with OXA 135 mg/sm, 4 patients were submitted to 3 PIPAC procedures, while 2 patients are still waiting for completing the treatment with the third procedure: to date no major complication was registered.

**Conclusions**: Cisplatin and doxorubicin may be safely used as repeated PIPAC at a dose of 30 mg/sm and 6 mg/sm, respectively; oxaliplatin can be used at an intraperitoneal dose of 135 mg/sm. Data will be confirmed after the completion of the treatment of all the patients enrolled. The efficacy analysis is in progress.

## ISSPP 2022 POSTER ABSTACT 41.

### OUTCOMES OF EMERGENCY PALLIATIVE SURGERY IN PATIENTS WITH PERITONEAL CARCINOMATOSIS

Xing-Yi Sarah Ong^1^, Tan, Joey Qiu Xuan^1^, Jane Seo^1^, Jolene Si Min Wong^1^, Claramae Chia^2^, Johnny Ong^3^

^1^National Cancer Centre Singapore; ^2^National Cancer Centre Singapore, Surgical Oncology; ^3^National Cancer Centre Singapore, Sarcoma, Peritoneal and Rare Tumours

**Background**: Peritoneal carcinomatosis (PC) is a marker of disease progression associated with poor prognosis that is often managed in the palliative setting. However, patients with PC can develop complications such as intestinal obstruction, perforation and intra-abdominal bleeding which may require lifesaving emergent surgical intervention. Emergency palliative surgery is rare and usually performed by a general surgical team, but is controversial as it is associated with high rates of post-operative morbidity and mortality.

We performed a retrospective cohort study to compare outcomes of patients with PC versus patients with colorectal cancer without peritoneal involvement (CRC) who underwent emergency surgery by a subspecialty team proficient in both curative and palliative management of peritoneal disease.

**Methods**: Patients who underwent non-elective surgery for complications of PC or CRC at a tertiary institution between 1 September 2019 and 31 October 2021 were identified by retrospective chart review. Data for outcomes including post-operative length of stay, complications (Clavien-Dindo III and above) and 30-day mortality was extracted for both groups. Statistical significance was assessed using Fisher’s exact test, with p-values less than 0.05 considered significant.

**Results**: A total of 40 patients were identified, with 17 patients in the PC group and 23 patients in the CRC group. Median post-operative length of stay in days was 13 in the PC group (IQR: 11-30) and 11 in the CRC group (IQR: 8-19.5). There were no patients with complications in the PC group and 1 (4.35%) in the CRC group, with no statistically significant difference between groups (p=1.00). There was 1 (5.88%) patient with 30-day mortality in the PC group and none in the CRC group, with no statistically significant difference between groups (p=0.425).

**Conclusion**: Post-operative morbidity and mortality outcomes for emergency surgery in patients with PC were comparable to that of patients with CRC when done by a subspecialty team. However, this study is limited by small sample size; further investigations are needed to characterize risks and benefits of emergency palliative surgery more definitively.

## ISSPP 2022 POSTER ABSTRACT 42.

### THE USE OF NATURAL LANGUAGE PROCESSING FOR REGISTRY DEVELOPMENT IN PERITONEAL SURFACE MALIGNANCIES

Louis Choon Kit Wong^1,#^, Nicholas Brian Shannon^1,#^, Chin-Ann Johnny Ong^1,2^, Chin Jin Seo^1^, Claramae Shulyn Chia^1^, Jolene Si Min Wong^1^

^1^Department of Sarcoma, Peritoneal and Rare Tumours (SPRinT), Division of Surgery and Surgical Oncology, National Cancer Centre Singapore, Singapore; ^2^Laboratory of Applied Human Genetics, Division of Medical Sciences, National Cancer Centre Singapore, Singapore

^#^Equal contributions

**Background**: In establishing a registry to augment research efforts in uncommon conditions such as peritoneal surface malignancies (PSM), traditional methods are often hindered by poor patient accrual and need for significant resources. We developed a novel pipeline using natural language processing (NLP) to accelerate this process and demonstrate its real-world application in identifying referral patterns of PSM patients for quality improvement purposes.

**Methods**: A training set comprising 100 radiological reports of abdomen and pelvis computed tomography scans was used to develop a rule-based NLP system able to classify reports based on the presence or absence of PSM. The algorithm was applied to a test set of 10,261 reports gathered at our institution from January to December 2021 to identify all patients with PSM for the creation of a PSM registry, which was subsequently populated using clinical records processed with an inhouse platform. These records were then analysed and referral patterns identified.

**Results**: The algorithm identified 251 reports as positive for PSM from a total of 10,261 reports. 239 were concordant with manual review, giving an incidence of 2.33 cases per 100 reports. Performance was excellent with a specificity of 90%, positive predictive value of 95%, accuracy of 96%, and Kappa of 0.91. From these, 228 unique patients were identified for registry inclusion. 27.6% of them were found to have been reviewed by the department of surgical oncology, while 72.3% were either not referred or referred late. 39.4% of patients not promptly reviewed were managed by medical oncology, 11.5% by colorectal surgery, 7.3% by gastroenterology, and 5.4% by internal medicine.

**Conclusion**: NLP is a useful tool in automated pipelines that can greatly contribute to registry creation and quality improvement efforts.

## ISSPP 2022 POSTER ABSTRACT 43.

### NABPACLITAXEL PRESSURIZED INTRA-PERITONEAL AEROSOL CHEMOTHERAPY COMBINED WITH SYSTEMIC NABPACLITAXEL-GEMCITABINE CHEMOTHERAPY FOR PANCREATIC CANCER PERITONEAL METASTASES: PROTOCOL OF SINGLE-ARM, OPEN-LABEL, PHASE II TRIAL (NAB-PIPAC TRIAL)

Andrea Di Giorgio^1^; Federica Ferracci^1^, Claudio Lodoli^1^, Francesco Santullo^1^, Miriam Attalla El Halabieh^1^, Carlo Abatini^1^,Serena Molica^1^, Cinzia Bagalà^2^, Pacelli, Fabio^1^

^1^Fondazione Policlinico Universitario Agostino Gemelli IRCCS, Peritoneum and Retroperitoneum Surgical Unit; ^2^Fondazione Policlinico Universitario Agostino Gemelli IRCCS, Medical Oncology

**Introduction**: Currently available therapies are poorly effective against peritoneal metastases (PM) of pancreatic origin. Pressurized Intra-Peritoneal Aerosol Chemotherapy (PIPAC) emerged as a novel intraperitoneal drug-delivery system. Recently, Van de Sande published a dose-escalation study identifying the safe dose of PIPAC administrated NabPaclitaxel. This drug is an ideal candidate for intraperitoneal chemotherapy and is highly active against pancreatic cancer cells. The combination of systemic NabPaclitaxel-Gemcitabine and NabPaclitaxel-PIPAC could offer better disease control to patients affected by pancreatic cancer with PM.

**Methods**: The Nab-PIPAC trial is a monocentric, prospective, open-label, phase II study (ClinicalTrials.gov identifier:NCT05371223). The primary objective of the study is to assess the antitumor activity of the combined treatment in terms of Disease Control Rate (DCR) according to the RECISTv.1.1 criteria. The secondary objectives include the assessment of feasibility, safety, pathological tumor response, progression-free and overall survival, the nutritional assessment, the Quality of Life, the NabPaclitaxel-PIPAC pharmacokinetics and PM molecular evolution with translational research.

Patients are scheduled for three combined treatment courses, each consisting of two cycles of endovenous NabPaclitaxel-Gemcitabine and one NabPaclitaxel-PIPAC(*Table*). NabPaclitaxel-Gemcitabine is administered according to the standard doses for metastatic pancreatic cancer, while intraperitoneal NabPaclitaxel is given at the dose of 112.5mg/m2. Simon’s two-stage design was used for sample size calculation, with 12 patients enrolled in the first stage and 26 in the second one (power80%, alpha0.1).

**Results**: Partial results will be available after first stage enrollment.

**Discussion**: This trial is intended to assess if NabPaclitaxel-PIPAC combined with systemic NabPaclitaxel-Gemcitabine has antitumor activity and it’s safe for pancreatic cancer patients with PM.

## ISSPP 2022 POSTER ABSTRACT 44.

### COMBINE HYPERTHERMIC INTRAPERITONEAL CHEMOTHERAPY (HIPEC) WITH CYTOREDUCTION SURGERY (CRS) AS CONVERSION SURGERY STRATEGY FOR LOCALLY ADVANCED DISTAL PANCREATIC CANCER AND THOSE WITH PERITONEAL METASTASIS

Shih-min Yin ^1^, Yueh-Wei Liu^1^, Sheng-En Chou^1^, Yu-Yin Liu^1^

^1^Department of General Surgery, Kaohsiung Chang Gung Memorial Hospital and Chang Gung University College of Medicine, Kaohsiung, Taiwan

**Introduction**: Herein, we report our experience of combine HIPEC with CRS and radical antegrade modular pancreatosplenectomy (RAMPS) for patients with distal pancreatic adenocarcinoma (PDAC) with peritoneal carcinomatosis or locally advanced tumor.

**Methods**: From 2019-2022, we retrospective review PDAC patients with peritoneal carcinomatosis or unresectable locally advanced tumor underwent neoadjuvant chemotherapy followed by conversion surgery.

**Results**: Totally 7 patients underwent conversion surgery after complete neoadjuvant chemotherapy. 3 patients with residual peritoneal carcinomatosis underwent HIPEC with CRS and RAMPS. Gemcitabine 1600mg was used in 1 case and cisplatin 150mg was use in 2 cases during HIPEC. The mean PCI score was 15.25, and CC0 was achieve in all 3 cases. For other four patients without peritoneal carcinomatosis, 2 patients received RAMPS only and 2 patients received RAMPS plus celiac axis resection (CAR) during the conversion surgery. Six of seven patients received adjuvant chemotherapy with gemcitabine-base regimen postoperatively. Until submission of this abstract, 3 patients underwent HIPEC+CRS survived without evidence of recurrence for more than 15 months. However, other 4 patients without HIPEC had recurrence within 6 months and all had mortality within 12 months.

**Conclusion**: HIPEC with CRS may be considered a treatment option for PDAC patients peritoneal carcinomatosis as conversion surgery. This option might be considered to expended to those patients with locally advanced PDAC as well.

## ISSPP 2022 POSTER ABSTRACT 45.

### CREATION, IMPLEMENTATION AND EARLY EXPERIENCE WITH CYTOREDUCTIVE SURGERY-SPECIFIC ENHANCED RECOVERY AFTER SURGERY PLAN IN A HIGH-VOLUME CENTER

Lana Bijelic^1^, Domenico Sabia^1^, Marina Bosch Ramirez^1^, Idoia Bonet, Ruth Lopez^1^

^1^Hospital de Sant Joan Despi Moises Broggi, Surgery

**Introduction**: Enhanced recovery after surgery (ERAS) pathways have been demonstrated to offer benefits in colorectal surgery but wide adoption in more extensive open abdominal surgery is still developing. Cytoreductive surgery (CRS) for peritoneal metastases is a uniquely complex abdominal procedure and few data exist on implementation and impact of ERAS. We sought to developed and apply a CRS-specific, tiered postoperative ERAS pathway in a high-volume referral center for peritoneal surface oncology.

**Methods**: The pathway was developed by a multidisciplinary group involving surgeons, anesthesiologists, nutritionists and nurses in 3 rounds of consultations. Specific care steps and recovery expectations were defined in the areas of physical activity, oral intake, management of drains and pain control. The pathway was activated on May 1, 2022, and all patients undergoing CRS are included prospectively. We created a tiered system with three distinct pathways based on complexity of intervention.

Pathway 1 is low complexity and characterized by no ICU admission, avoidance of nasogastric (NG) tube, no parenteral feeding with oral intake and ambulation on day 1.

Pathway 2 is intermediate complexity characterized by early (day 2-4) NG removal, epidural de-escalation and early mobilization.

Pathway 3 is high complexity characterized by classic management (4 drains, NG removal day 4-6, 5-day epidural and ambulation on day 3).

A document describing specific daily steps for each pathway was created and distributed in all areas of recovery (ICU, post-anesthesia unit and floor). A checklist for documentation of nursing tasks and a data collection tool for physicians was implemented. Prospective inclusion will allow for outcomes assessment in approximately 40 patients by September 2022 with measurement of compliance and impact on morbidity and length of stay.

## ISSPP 2022 POSTER ABSTRACT 46.

### LIQUID BIOPSY OF THE PARACRINE MICROENVIRONMENT FOR PC: PROGNOSTICATION, THERAPY AND PATIENT ACCEPTANCE

Gillian Ng^1^, Hongyuan Zhu^2^, Ying Liu^2^, Qiu Xuan Tan^2^, Joey Tan^2^, Wai Har Ng^1^, Josephine Hendrikson^1^, Hui Jun Lim^1^; Irene W.Tu^1^, Jolene Si Min Wong^1^, Yee Pin Tan^1^, Irene E. Teo^1^, Jimmy Bok Yan So^3^, Claramae Chia^4^, Johnny Ong^1^;

^1^National Cancer Centre Singapore, Department of Sarcoma, Peritoneal and Rare Tumours (SPRinT); ^2^National Cancer Centre Singapore; ^3^National University Hospital, Singapore; ^4^National Cancer Centre Singapore, Surgical Oncology

**Introduction**: Current therapeutic strategies for peritoneal carcinomatosis (PC) have not exploited the reliance of tumour cells on ascites. We recently demonstrated that targeting key paracrine factors in the PC fluid microenvironment is therapeutically efficacious. This study thus aimed to tailor appropriate management of PC patients through the expectation and perspectives of novel prognostic and potentially therapeutic biomarkers amongst patients and their caregivers.

**Methods**: Using multi-omics analysis and independent validation in 105 patients, we demonstrated the prognostic significance of a 3-biomarker panel. 30 pairs of patients and their caregivers were interviewed through a 15-min questionnaire, which was created specifically for our local clinical climate, to determine their receptivity towards the panel.

**Results**: The clinical prognostic effect of a 3-biomarker panel was validated in PC patients’ ascites (*p*<0.0001). 83.3% of respondents were receptive to the panel if it had a 90% accuracy rate, considering the relative immensity in their decision-making process on pursuing palliative surgery. Caregivers’ acceptance of the panel was intertwined with patients’ autonomy. 93.3% of them gave a score of 3 or more on a scale of 5 on feeling in control of disease progression via awareness of the panel results. 61.6% of respondents agreed that they wished to be informed of panel results regardless of the outcome. Two recurring themes (cost and emotional stress) could prevent the panel’s use. 40% were keen to pay only up to S$300 (∼205€) while 48.3% preferred not to pay. 23% resonated that knowing too much may cause them to overthink, even if it enlists better care management.

**Conclusions**: Our team identified a 3-biomarker panel that accurately prognosticated PC patients. Our study suggests sound translational research by incorporating patient-caregiver preference, thereby allowing the tailoring of appropriate management of PC patients.

## ISSPP 2022 POSTER ABSTRACT 47.

### ATTITUDES, KNOWLEDGE & AWARENESS AMONGST PHYSICIANS IN THE MANAGEMENT OF PERITONEAL SURFACE BASED MALIGNANCIES

Irene Ng^1^, Benjamin Paik^2^, Jolene Si Min Wong^3^, Johnny Ong^4^, Jane Seo^3^, Piea Peng Lee^3^, Darryl Juan^5^, Hongyuan Zhu^3^, Claramae Chia^6^

^1^National Cancer Centre Singapore; Singapore General Hospital; ^2^Lee Kong Chian School of Medicine; ^3^National Cancer Centre Singapore; ^4^National Cancer Centre Singapore, Sarcoma, Peritoneal and Rare Tumours; ^5^Singapore General Hospital; ^6^National Cancer Centre Singapore, Surgical Oncology

**Introduction:** Peritoneal surface-based malignancies (PSM) represent a diverse group with a spectrum of disease biologies. Despite growing evidence of cytoreductive and hyperthermic intraperitoneal chemotherapy (CRS-HIPEC) as the standard treatment in selected PSM patients, it is underutilized amongst physicians. Delayed or missed referrals are common due to unfamiliarity amongst the general physician population on its clinical presentation and treatment options. This study aims to evaluate the level of knowledge and awareness of PSM amongst physicians and determine trends in referral patterns and barriers to early referrals.

**Methods:** A cross-sectional online survey was conducted across primary and tertiary healthcare centres nationwide. The survey comprised of 3 sections: (i) assessing clinical experience and demographics, (ii) knowledge and awareness of PSM, (iii) referral patterns and barriers for early referrals. Total aggregate scores were derived and compared.

**Results:** 91 complete responses were obtained. The majority of the participants (52%) reported unfamiliarity with PSM assessment tools and treatment modalities. Mean total aggregate knowledge score was 8.05 out of 14 (95% CI 7.2 - 8.9). Domain scores in appendiceal, colorectal and ovarian malignancies were 1.8, 1.8 and 1.7 out of 3 respectively, with worst performance in PSM secondary to ovarian malignancies. Physicians who were unaware of any local peritoneal specialist unit were more likely to have lower aggregate scores (p= 0.023). A lack of a seamless referral system and perceived high morbidity and mortality of cytoreductive and hyperthermic intraperitoneal chemotherapy (CRS-HIPEC) surgeries were significant barriers for referrals.

**Conclusion:** There is a lack of knowledge on PSM and its treatment amongst the general physician population, resulting in delayed referrals and treatment. Misconceptions need to be corrected urgently. Awareness on the efficacy and safety profile of current treatment modalities must be improved. Lastly, a seamless primary-tertiary referral system should be implemented to facilitate timely referral.

## ISSPP 2022 POSTER ABSTRACT 48.

### PIPAC IN CANCERS OF THE COLON, OVARY AND STOMACH (THE PICCOS TRIAL)

Sadie Jones^1^, Jamie Murphy^2^, Sarah Gwynne^3^, Richard Adams^4^, Christopher Peters^5^, Emma Hudson^6^, Jonathan Frost^7^, Jane Blazeby^8^, Nixon, Lisette^9^, Leona Batten^10^, Angela Casbard^10^, Jared Torkington^11^

^1^University Hospital of Wales Healthcare NHS Trust, Obstetrics and Gynaecology; ^2^Imperial College London; ^3^Singleton Hospital, Oncology; ^4^Velindre University NHS Trust, Oncology; ^5^Imperial College London, Surgery; ^6^Velindre University NHS Trust, Oncology; ^7^Royal United Hospitals Bath NHS Foundation Trust; ^8^University of Bristol; ^9^Cardiff University, Centre for Trials Research; ^11^University Hospital of Wales Healthcare NHS Trust, Surgery

**Introduction**: In 2021 the National Institute for Clinical Excellence (NICE) published interventional procedures guidance that stipulated that in the UK, Pressurised IntraPeritoneal Aerosolised Chemotherapy (PIPAC) should only be used within the context of a randomised control trial to demonstrate efficacy against standard of care. The UK PIPAC collaborative would like to present its first randomised controlled trial assessing the efficacy of PIPAC in the management of peritoneal metastases (PM) in patients with cancer of the colon, ovary and stomach. The PICCOS trial aims to not only assess efficacy compared to standard of care in terms of progression free survival (PFS), but also quality of life.

**Methods**: This is a basket, phase II trial with a master protocol covering the overarching research methodology, and embedded individual cancer site specific protocols, sample sizes and analysis plans. 78 patients with PM from colon cancer, 62 patients with PM from ovarian cancer and 72 patients with PM from stomach cancer will be randomised to systemic chemotherapy or alternating PIPAC and systemic chemotherapy every 2 or 3 weeks for 18 weeks in total. The primary outcome measure is PFS. CT scans undertaken every 8 weeks following treatment will be assessed against the RECIST criteria to assess disease burden and determine PFS. Quality of life will be assessed using the EORTC QLQ C30 tool.

**Results**: The PICCOS Trial has now secured funding with a National Institute of Health Research Efficacy and mechanism Evaluation (NIHR EME) grant and is due to commence in November 2022 with a four-year running period. Publication and dissemination of results is anticipated in 2027.

**Conclusions**: This is the first UK randomized controlled trial assessing the efficacy and impact of quality of life of PIPAC in the treatment of peritoneal metastases aiming to provide high quality evidence to guide clinical practice and further research in the future.

## ISSPP 2022 POSTER ABSTRACT 49.

### HISTONE DEACETYLASE INHIBITOR VALPROIC ACID AS A PREVENTION OF PERITONEAL GASTRIC CANCER RECURRENCE

^1^Brauer, Lisa

^1^ University of Tübingen, for General, Visceral and Transplant Surgery

**Introduction**: VPA is a histone deacetylase inhibitor that exhibits wide-ranging effects on cancer hallmarks and is a potential therapeutic agent in PIPAC to prevent peritoneal gastric cancer recurrences.

**Methods**: Our experiments examined the effect of HDAC inhibition of VPA, in combination or not with PTX, on tumor features in-vitro, compared to a control group (NaCl 0.9%). The treatment was applied under PIPAC conditions. The cell number of living MKN-45 was measured over one week to analyze the cytotoxic effect of the drugs (CASY assay). The metabolic effect of VPA and PTX on vital MKN-45 was examined with an MTT assay. A scratch assay was determined to investigate the migration of treated MKN-45 cells and the wound healing of drug-treated fibroblasts (NHDF) and mesothelial cells (MeT-5A). The adhesion of MKN-45 cells was tested by an adhesion assay with ECM protein-coated plates.

**Results**: The CASY assay resulted only in a slow-acting and minimal cytotoxic effect of VPA alone on MKN-45 cells. The different concentration groups of VPA showed hardly any differences in cell growth among them. The metabolic effect of VPA depended on the concentration level. From a concentration of 2.02 mg/ml VPA, an immediate metabolic inhibition was induced. In the scratch assay, all the different concentrations of VPA produced an immediate inhibition of gastric cancer cell migration. The wound-healing assay with normal NHDF and MeT-5A cells treated with VPA alone documented no wound closure. VPA alone inhibited cell adhesion. One combination of VPA and PTX showed a synergistic cytotoxic and metabolic effect on MKN-45 cells.

**Conclusion**: VPA could be applied with PIPAC technology but seems to be not implicated in preventing intraperitoneal gastric cancer recurrence without impairing wound healing. VPA has inhibitory effects on all analyzed gastric cancer hallmarks in a concentration-dependent manner. The chemosensitizing effect of PTX and VPA depended on the analyzed cancer hallmark and drug concentration.

## ISSPP 2022 POSTER ABSTRACT 50.

### THE USE OF PRESSURIZED INTRA-PERITONEAL AEROSOLIZED CHEMOTHERAPY (PIPAC) FOR BREAST PERITONEAL METASTASES: CASE SERIES & REVIEW OF LITERATURE

Jasmine Chang^1^, Louis Wong^2^, Jane Seo^2^, Darryl Juan^3^, Johnny Ong^4^, Claramae Chia^5^, Wong, Jolene^2^

^1^National Cancer Centre Singapore, General Surgery; ^2^National Cancer Centre Singapore; ^3^Singapore General Hospital, SPRinT; ^4^National Cancer Centre Singapore, Sarcoma, Peritoneal and Rare Tumours; ^5^National Cancer Centre Singapore, Surgical Oncology

**Introduction:** Breast cancer is common and is a leading cause of cancer death amongst women despite advances in treatment modalities. Breast peritoneal metastasis (BPM)are rare and portend a dismal prognosis; reported median survival were 5.8 versus 22.6 months in those with other metastatic sites. Current therapies such as chemo-, immuno- and hormonal therapy remain unsatisfactory due to their poor penetration into the peritoneal cavity. PIPAC is a promising novel locoregional therapy found to be efficacious in gastrointestinal and gynecological PM. However, its use in BPM is not well studied. Hence we aim to evaluate our institutional experience with BPM and perform a systematic review of literature on the utility of PIPAC in this disease entity.

**Methods:** Review of a prospective maintained PIPAC database followed by a systematic review of literature from PubMed and Embase databases.

**Results:** Two patients underwent PIPAC for BPM. They were diagnosed with IDC and ILC respectively and suffered metachronous peritoneal recurrence more than 5 years after primary cancer diagnosis. Both experienced progressive disease and increasing symptoms from PM despite an average of 2 lines of systemic therapy. PIPAC was offered an alternative. Median PCI score was 24.5; Doxorubicin 1.5mg/m^2^/Cisplatin 7.5mg/m^2^ (D/C) and Abraxane 85mg/m^2^ regimes were given. There was no adverse response and mean quality of life (QoL,FACT-G) scores saw a more than 2-fold increment post-PIPAC.

In our systematic review, 31 patients underwent 402 PIPAC procedures over a median follow up of 5 months. The most commonly agent used was D/C, median PCI amongst BPM patients was 16.6 and a median of 2.2 PIPACs were performed prior to disease progression. Median OS was 11.7months. All studies demonstrated an improvement in PCI and ascites volume while improving QOL.

**Conclusion:** PIPAC is a novel treatment strategy for BPM and is associated with good tolerability and improved QoL outcomes.

## ISSPP 2022 POSTER ABSTRACT 51.

### ALBUMIN REPLACEMENT PROTECTS RENAL FUNCTION IN PATIENT UNDERGOING CYTOREDUCTIVE SURGERY AND CISPLATIN-BASED HYPERTHERMIC INTRAPERITONEAL CHEMOTHERAPY

Huang, Szu Wei^1^

^1^Chang Gung Memorial Hospital Kaohsiung Branch, Obstetrics and Gynecology

**Introduction**: The aim of our study was to reveal the efficacy of the use of perioperative albumin replacement for preventing acute kidney injury (AKI) after cytoreductive surgery (CRS) with cisplatin-based hyperthermic intra-peritoneal chemotherapy (HIPEC).

**Methods**: We retrospectively enrolled patients with gynecological malignancy who received CRS followed by cisplatin-based HIPEC from July 2018 to July 2021 in our hospital. Our strategies to prevent post-operative AKI included achieving urine output goal over 1 mL/kg/h and administration of albumin replacement (human albumin 20% infusion) since initiation of CRS until at least one day after surgery. We analyzed the incidence of postoperative AKI which is defined as a glomerular filtration rate (GFR) on the seventh day after surgery 25% lower than on the first day after surgery. The GFR was estimated by the Cockcroft-Gault formula.

**Results**: A total of 29 cases were analyzed. The median cisplatin dose was 95mg/m2 (range from 50 to 120 mg/m2). The median preoperative serum albumin was 4.2 g/dL (range from 2.4 to 4.7 g/dL). Only one patient had a decrease in GFR more than 25%. The incidence of postoperative AKI in this cohort was 3.45% (1/29) which is relatively low compared to previous data.

**Conclusions**: The perioperative fluid management to achieve adequate urine output combined with albumin infusion is an effective strategy to prevent AKI after CRS and cisplatin-based HIPEC.

## ISSPP 2022 POSTER ABSTRACT 52.

### Achieving Intraperitoneal Disease Control using Cytoreductive Surgery and Hyperthermic Intraperitoneal Chemotherapy: Two Cases of Metastatic Breast Cancer

Philipp Barakat^1^, Mary Caitlin King^1^, Andrei Nikiforchin^1^, Luis Felipe Falla Zuniga^1^, Felipe Lopez-Ramirez^1^, Carol Nieroda^1^, Vadim Gushchin^1^, Armando Sardi^1^

^1^Mercy Medical Center, U.S.A

**Introduction**: Peritoneal metastases (PM) of breast cancer tend to occur late in the disease course and are challenging to manage with unpredictable response to chemotherapy and often significant symptoms. Since presentation and complications are similar to ovarian and appendiceal cancers with PM, cytoreductive surgery and hyperthermic intraperitoneal chemotherapy (CRS/HIPEC) may be an option to address resistant peritoneal disease in these patients. We assessed intraperitoneal disease control and perioperative outcomes in 2 cases of PM from breast cancer treated with CRS/HIPEC.

**Methods**: We report 2 female patients with PM from breast cancer treated with CRS/HIPEC. Perioperative variables and outcomes are described. Adverse events within 90-days of CRS/HIPEC were categorized according to CTCAE v5.

**Results**: Patient 1, diagnosed at age 64, had hormone positive/HER2 negative lobular carcinoma treated with mastectomy, chemotherapy, and hormonal therapy. Prior to salvage CRS/HIPEC at age 72, 5 cycles of intraperitoneal chemotherapy via indwelling catheter failed to control recurrent peritoneal disease. Patient 2, diagnosed at age 52, had hormone positive/HER2 negative ductal-lobular carcinoma and received lumpectomy, hormonal and target therapy. Prior to salvage /HIPEC at age 59, she had frequently recurring ascites, resistant to hormonal therapy and requiring multiple paracenteses. Both had complete CRS/HIPEC with melphalan. Major complication was anemia that required transfusion in both patients. No other major morbidity occurred, and they were discharged on postoperative day 8 and 13, respectively. Patient 1 had peritoneal recurrence 26 months post-CRS/HIPEC and died of disease at 49 months. Patient 2 never had peritoneal recurrence but died of extraperitoneal progression at 38 months.

**Conclusion**: PM of breast cancer can cause debilitating symptoms and creates unique challenges for oncologists. CRS/HIPEC is safe and can provide intraperitoneal disease and symptom control in these select patients. This case report may allow clinicians to consider CRS/HIPEC for these rare and challenging patients.

**Table 1**: Perioperative Characteristics by Patient.

**Table:** 

	Patient 1	Patient 2
Age at Diagnosis, years	64	52
Age at CRS/HIPEC, years	72	59
Stage	T1cN2M0, IIIa (AJCC7)	T1N0M0, Ia (AJCC7)
Pre-CRS/HIPEC Histology	Lobular carcinoma, ER/PR positive, HER2 negative	Ductal-lobular carcinoma, ER positive, PR/HER2 negative
Post-CRS/HIPEC Histology	Lobular carcinoma, ER/PR positive, HER2 negative 0/11 LN positive	Ductal-lobular carcinoma, ER/PR positive, HER2 negative, 0/13 LN positive
Treatment before CRS/HIPEC	Multiple^a^	Multiple^b^
Prior Surgical Score	0	1
PCI at Exploration	14	29
PCI Post-CRS/HIPEC	4	3
CC-Score	CC-1	CC-1
Site of Residual Disease	Small bowel mesentery (scarring)	Small bowel mesentery (scarring)
Number of Major Resections	5^c^	7^d^
HIPEC Agent	Melphalan	Melphalan
Length of ICU Stay, days	1	1
Length of Stay, days	8	13
Complications (CTCAE v.5)	Anemia (G3), Thrombocytopenia (G1)	Anemia (G3), Thrombocytopenia (G2), Oral thrush (G2)
Adjuvant Therapy	Raloxifene	Letrozole + palbocilib
Initial Site of Recurrence	Rising tumor markers	Liver parenchyma
Progression-free Survival, months	5	2
Time to Peritoneal Recurrence, months	26	none
Status	DOD	DOD
Overall Survival, months	49	38

^a^ Right mastectomy with no adjuvant treatment; Left mastectomy with radiation and chemotherapy with docetaxel/cyclophosphamide; Letrozole x10 months; Tamoxifen x2.5 years; Hemicolectomy for colon metastasis followed by exemestane; Fulvestrant x4 months; Intraperitoneal cisplatin x5 cycles. ^b^ Right lumpectomy with radiation; Anastrazole x2 months; Letrozole/palbociclib x6 months and restarted after CRS/HIPEC. ^c^ Splenic flexure, previous ileocolonic anastomosis, omentectomy including the gastroepiploic arcade, resection of liver capsule segments 2-6, right and left diaphragmatic peritonectomy. ^d^ Bilateral parietal peritonectomy, right diaphragmatic peritonectomy, resection of liver capsule segments 1 and 4-8, portal dissection, omentectomy, splenectomy, hysterectomy, bilateral salpingo-oophorectomy. Overall and progression-free survival are calculated from the date of CRS/HIPEC to date of death and date of initial recurrence, respectively. AJCC – American Joint Committee on Cancer; CC-Score – completeness of cytoreduction score; CRS/HIPEC – cytoreductive surgery with intraperitoneal chemotherapy; CTCAE – Common Terminology Criteria for Adverse Events; DOD – dead of disease; ER – estrogen receptor; G – grade; HER2 – Human epidermal growth factor receptor 2; ICU – intensive care unit; LN – lymph node; PCI – peritoneal cancer index; PR –progesterone receptor.

## ISSPP 2022 POSTER ABSTRACT 53.

### HIGH INTRA-ABDOMINAL PRESSURE DURING HYPERTHERMIC INTRAPERITONEAL CHEMOTHERAPY (HIPEC) FOLLOWING CYTOREDUCTIVE SURGERY (CRS) FOR PERITONEAL SURFACE MALIGNANCIES

Louis Choon Kit Wong^1,#^, Jolene Si Min Wong^1,#^, Chin Jin Seo^1^, Khee Chee Soo^1^, Chin-Ann Johnny Ong^1,2^, Claramae Shulyn Chia^1^

^1^Department of Sarcoma, Peritoneal and Rare Tumours (SPRinT), Division of Surgery and Surgical Oncology, National Cancer Centre Singapore, Singapore; ^2^Laboratory of Applied Human Genetics, Division of Medical Sciences, National Cancer Centre Singapore, Singapore

^#^Equal contributions

**Background**: Cytoreductive surgery (CRS) and hyperthermic intraperitoneal chemotherapy (HIPEC) represent a mainstay of treatment for peritoneal malignancies. There is evidence that HIPEC using high intra-abdominal pressure (IAP) results in increased tissue penetration, although its safety profile remains relatively unknown. We thus aim to evaluate differences in intra- and post-operative outcomes in patients undergoing CRS-HIPEC with different levels of IAP.

**Methods**: This prospective cohort study was conducted from January 2020 to February 2021 with patients undergoing CRS-HIPEC. Low IAP during HIPEC was defined as <18 mmHg and high IAP as ≥18 mmHg. Data was collected on patient and tumour characteristics, intra-operative clinical and biochemical parameters, and immediate postoperative outcomes.

**Results**: 40 patients underwent CRS-HIPEC (n low=20, n high=20). Median IAP in the low and high IAP groups were 12.0 and 19.0 mmHg respectively. During HIPEC, both groups experienced increase in heart rate, central venous pressure, end tidal CO2 (ETCO2), temperature, and serum glucose, with decrease in mean arterial pressure and base excess. There were no significant differences in hemodynamics between the 2 groups. Mild electrolyte derangements and a decrease in hemoglobin were noted in the high IAP group but were of small magnitude. Post-operatively, high IAP did not result in increased rate of complications, time to full feeds, ICU or total hospital stay.

**Conclusions**: High IAP in HIPEC is well tolerated and did not result in additional adverse events.

## ISSPP 2022 POSTER ABSTRACT 54.

### THE UTILITY OF AN ERAS PROGRAM IN CRS HIPEC PATIENTS

Jing Ting Wu^1^, Jane Seo^2^, Jolene Si Min Wong^2^, Johnny Ong^3^, Claramae Chia^4^

^1^Singapore General Hospital; ^2^National Cancer Centre Singapore; ^3^National Cancer Centre Singapore, Sarcoma, Peritoneal and Rare Tumours; ^4^National Cancer Centre Singapore, Surgical Oncology

**Introduction**: Benefits of enhanced recovery after surgery (ERAS) protocol have been well documented to improve patient outcomes. The basic tenets of ERAS include the adoption of evidence-based practices to decrease surgical stress, maintain physiologic homeostasis and facilitate the recovery of patients. Improved outcomes with ERAS implementation in colorectal and gynaecological surgeries have been well documented in large-scale randomised controlled trials and structured nationwide programs.

**Aims:** This study aims to show that the success of ERAS can be replicated in multi-visceral resections in patients undergoing cytoreductive surgery and hyperthermic intraperitoneal chemotherapy (CRS-HIPEC), with the primary endpoint being a reduction in length of hospital stay (LOS) and secondary endpoints being similar or reduced 30-day readmission rates and return to bowel activity.

**Methods**: This is a prospective study with recruitment of patients with primary peritoneal disease or peritoneal-limited metastatic disease suitable for CRS-HIPEC in a high-volume surgical oncology unit. We formulated an ERAS protocol of 22 items comprising of surgical, anaesthesia and allied health interventions, divided into the preoperative, intra-operative, and post-operative period. Applying this protocol prospectively to a series of patients undergoing CRS-HIPEC in our institution, we analyse length of stay (LOS), morbidity rates, 30-day readmission rates, return to bowel activity, and patient satisfaction.

**Results**: A total of 12 eligible patients were recruited from the period of January 2022 to June 2022, with compliance to the ERAS protocol. Mean LOS was 11 days (± S.D. 5.2) in the ERAS population as compared to a mean LOS of 12.6 days (± S.D. 4.7) in the pre-intervention population in our institution. 30-day readmission rates were 16%, and return to bowel activity was on average on post-operative day 3 (± S.D. 1.2) for first flatus and postoperative day 4 (± S.D. 1.7) for first bowel output.

**Conclusion**: The use of ERAS in CRS-HIPEC is feasible, with shorter length of stay without compromising on 30-day readmission rates and complication rates.

## ISSPP 2022 POSTER ABSTRACT 55.

### IMPACT OF MACROSCOPIC TUMOR CONSISTENCY AND EPIC IN LOW-GRADE APPENDICEAL NEOPLASMS WITH PSEUDOMYXOMA PERITONEI

Raymond Hayler^1^, Ernest Cheng^1,2^, Raphael Shamavonian^1^, Jasmine Mui^1,2^, Josh Karpes^1^, Kerry Chen^1^, Nima Ahmadi^1^, David L. Morris^1,2^

^1^Liver and Peritonectomy Unit, Department of Surgery, St. George Hospital, Kogarah, Australia; ^2^St. George Hospital Clinical School, University of New South Wales, Kogarah, Australia

**Background**: Low-grade appendiceal mucinous neoplasm (LAMN) with pseudomyxoma peritonei (PMP), otherwise known as disseminated peritoneal adenomucinosis (DPAM), is treated with cytoreductive surgery (CRS) and hyperthermic intraperitoneal chemotherapy (HIPEC) with or without Early Postoperative Intraperitoneal Chemotherapy (EPIC). These tumors can vary significantly intraoperatively in terms of macroscopic appearance and consistency, and this may have an effect on survival outcomes. This study examines the differences between soft and hard tumours in terms of short-term outcomes and long-term survival and whether this can change with the use of EPIC.

**Patients and Methods**: Patients were classified into having macroscopically soft or hard tumours. A comparison of short-term outcomes (major morbidity, mortality, length of ICU stay and length of hospital stay) and long-term outcomes (overall survival) was performed. Survival sub analysis was performed in the patients who received EPIC.

**Results**: Soft tumors behaved differently, with lower morbidity, mortality, shorter ICU stay and hospitalization. They also demonstrated greater overall survival (86.7% at 5years, *p*<0.001). EPIC improved the overall survival for both soft and hard tumors.

**Conclusion**: Tumor consistency has an impact on the outcomes of patients with DPAM and this can be used for prognostication.

## ISSPP 2022 POSTER ABSTRACT 56.

### PRESSURIZED INTRAPERITONEAL AEROSOL CHEMOTHERAPY, REASONS FOR INCOMPLETE TREATMENT: SYSTEMATIC REVIEW OF LITERATURE

Anne-Cecile Ezanno ^1^, Brice Malgras ^1^, Marc Poacrd^2^

^1^Service de Chirurgie HIA Begin - Saint-Mandé (France), ^2^Service de Chirurgie La Pitié Salpétrière - Paris (France)

**Introduction**: Pressurized intraperitoneal aerosol chemotherapy (PIPAC) emerged in the last few years as a novel method of drug administration with encouraging results in the treatment of peritoneal metastasis (PM) of several origins. The current recommendations foresee at least 3 PIPAC. However, some studies have reported consistent failure rates from the second PIPAC (>15%). Some patients do not complete the full treatment course and have to stop after only 1 or 2 procedures, with probably hence limited benefit (6).

**Methods**: A literature review was performed according to the Preferred Reporting Items for Systematic Reviews and Meta-Analyses (PRISMA) guidelines: search terms included “PIPAC” and “pressurized intraperitoneal aerosol chemotherapy.” After reading the articles, we only analyzed those who relate to their causes of premature arrest for PIPAC treatment.

**Results**: The systematic search identified 30 published clinical articles concerning PIPAC and there reasons to stop PIPAC. The series range from 3 to 144 patients, with a total of 1289 patients treated with PIPAC for various tumors. A total of 2602 PIPAC treatments were administered. The median number of PIPAC treatments per patient was 2.3. The median PCI score at the time of the first PIPAC was 17 (14.6-20). The number of patients who don’t complete the recommended 3 PIPAC was 576 (44.7%), either median failure of completed treatment of 50% per study. Disease progression was the main reason to stop PIPAC treatment (18.4%). The other reasons to stop PIPAC earlier were death (1.4%), patient’s wish (4%), adverse events (2.2%), conversion to curative cytoreductive surgery (1.7%) and medical reason (emboly, pulmonary infection…) (5%). In patients who underwent PIPAC, morbidity median rate was 13% (mean 19.1±13.8) and the mortality median rate was 0% (mean 1.3± 3.2%).

**Conclusions**: Investigations are necessary to well know reason to interrupt PIPAC treatment and also improving selection of patients who are most likely to benefit PIPAC.

## ISSPP 2022 POSTER ABSTRACT 57.

### RARE CAUSE FOR PERITONEAL METASTASIS: STK11-ADNEXAL TUMOUR

Solass W^1^, Pagano F^2^, Saner F^2^, Imboden S^2^, Mueller MD^2^

^1^Department of Pathology, University of Berne, Berne, Switzerland; ^2^Department of Obstetrics and Gynecology, University Hospital of Berne and University of Berne, Berne, Switzerland

Corresponding author: wiebke.solass@unibe.ch

**Introduction**: Peritoneal metastasis are common in gynaecological pathology and mainly arise from ovarian neoplasms, especially high and low-grade serous ovarian cancer. The differential diagnosis includes the exclusion of other primary tumours in the gastrointestinal tract, breast neoplasms and should always be in correlation with the clinical and radiological image. However, there are rare cases that do not fall within the routine morphological and immunhistochemical patterns and should be evaluated carefully. Here we present a rare (22 described cases worldwide) and new entity in gynaecological pathology that can develop peritoneal metastasis and should be taken into consideration if clinical presentation, localisation, morphology and immunohistochemistry are non-conclusive.

**Case report:** We present a case of a 31-year-old woman with coincidentally diagnosed mass in the left adnexal loge during routine inspection. No prior history of disease and inconspicuous family history was documented. Transvaginal ultrasound and MRI of the abdomen/pelvin confirmed a 6.5 cm large mass in the left adnexal loge with potential origin from the fallopian tube. During laparoscopic resection, a well-circumscribed nodular tumor in the peri-adnexal soft tissue (ligament) was seen and resected. Additionally, three isolated nodules of the douglas peritoneum and the peritoneum of the sacrouterine ligament were send for histological analysis.

Pathological work-up was challenging due the heterogeneous growth pattern and unusual localization of the tumor. Standard immunohistochemistry was non-conclusive, showing a homogenous WT1 positivity, patchy PAX8 positivity, P53- Wildtype and only a moderate proliferation index of 10-20%. This pattern excluded a high-grade serous carcinoma and morphology did not match with a low-grade serous carcinoma. Due to the localization, other entities like Wolffian- neoplasms and sex cord stromal tumors were ruled out. After a thorough literature search a STK11 mutated adnexal neoplasm needed to be ruled and next-generation sequencing was performed, which detected a STK11 mutation (c.734+1G>A 86.3%) and concluded the diagnosis.

**Conclusion**: STK11 mutated adnexal tumor is new defined subtype of adnexal neoplasms which characterized by a variety of morphological growth patterns, non-conclusive immunohistochemistry and characteristic STK11 mutation. Furthermore, these neoplasms can be the origin of peritoneal metastasis. Currently, only 22 cases worldwide are described in the literature. Approximately 50% of these cases have an association with Peutz-Jeghers syndrome. The clinical outcome is variable depending on the completeness of the surgical resection. Metastasis and/or recurrence of disease is described in 80% of these 22 patients. It is important to raise the awareness of this new entity among clinicians and pathologists.

## ISSPP 2022 POSTER ABSTRACT 58.

### MULTICENTER DOSE-ESCALATION PHASE 1 TRIAL OF MITOMYCIN C PRESSURIZED INTRAPERITONEAL AEROSOLIZED CHEMOTHERAPY IN COMBINATION WITH SYSTEMIC CHEMOTHERAPY FOR APPENDICEAL AND COLORECTAL CARDINOMATOSIS

Mustafa Raoof^1^, Kevin Sullivan^1^, Paul Frankel^2^, Marwan Fakih^3^, Joseph Chao^3^; Dean Lim^3^, Yanghee Woo^1,4^, I. Benjamin Paz^1^, Yuman Fong^1^, Rebecca Thomas^5^, Sue Chang^6^, Melissa Eng^7^, Raechelle Tinsley^8^, Richard Whelan^9^, Danielle Deperalta^9^, Jeremy Jones^10^, Amit Merchea^11^; Thanh Hue Dellinger^1^

^1^City of Hope National Medical Center, Surgery; ^2^ City of Hope National Medical Center, Biostatistics; ^3^City of Hope National Medical Center, Medical Oncology; City of Hope Comprehensive Cancer Center, Beckman Research Institute; ^5^Northwell Health, Pathology; ^6^City of Hope National Medical Center, Pathology; ^7^City of Hope National Medical Center, Office of Clinical Research; ^8^City of Hope National Medical Center, Office of Clinical Research; ^9^Northwell Health, Surgery; ^10^Northwell Health, Medical Oncology; Mayo Clinic, Surgery; ^11^Mayo Clinic, Surgery

**Introduction**: Peritoneal carcinomatosis (PC) from appendiceal or colorectal cancer has significant morbidity and limited survival. Pressurized intraperitoneal aerosolized chemotherapy (PIPAC) is a promising minimally invasive approach to treat unresectable PC. This is a dose-escalation trial of mitomycin C (MMC)-PIPAC in combination with systemic chemotherapy.

**Methods**: In this US multicenter phase I study of MMC-PIPAC, patients who are ineligible for cytoreduction after at least 4 months of first- or second-line systemic chemotherapy will be included. Patients will be excluded if they have: progressed or intolerant of irinotecan-based chemotherapy; extraperitoneal metastases; bowel obstruction; or poor performance status (ECOG>2). Escalating doses of MMC-PIPAC (7 to 25 mg/m^2^) will be administered in combination with standard dose systemic FOLFIRI (Fig 1). With a 3+3 design over 4 dose levels, the safety evaluation will take 15 patients during the dose escalation and an additional 6 expansion patients (Total 21).

**Results**: The primary endpoint is to assess the safety of MMC-PIPAC in combination with FOLFIRI and to establish the recommended dose of MMC to be used for Phase 2 trials. Secondary endpoints include assessment of response by histology (peritoneal regression grade score), imaging (RECIST 1.1), and laparoscopy (peritoneal carcinomatosis index). Additional secondary endpoints include progression free survival, overall survival, technical failure rate, surgical complications, conversion to curative-intent cytoreduction, patient-reported outcomes, and functional status. Longitudinal blood and tissue specimens will be collected for translational correlatives including pharmacokinetics, circulating biomarkers, immune profiling, and single-cell transcriptomics.

**Conclusions**: This Phase 1 trial will establish the recommended Phase 2 dose of MMC-PIPAC when given in combination with FOLFIRI. We expect to detect an early efficacy signal supporting further development of this combination.

## ISSPP 2022 POSTER ABSTRACT 59.

### SAFETY AND EFFICACY OF OXALIPLATIN PRESSURIZED INTRAPERITONEAL AEROSOLIZED CHEMOTHERAPY IN APPENDICEAL AND COLORECTAL CANCER PATIENTS WITH PERITONEAL CARCINOMATOSIS: A FIRST-IN-US PHASE 1 STUDY

Mustafa Raoof^1^, Richard Whelan^2^, Kevin Sullivan^1^, Paul Frankel^3^, Marwan Fakih^4^, Joseph Chao^4^; Dean Lim^4^, Yanghee Woo^1,5^, I. Benjamin Paz^1^, Michael Lew^6^, Lorna Rodriguez-Rodriguez^1^, Yuman Fong^1^, Wiebke Solass^7^, Rebecca Thomas^8^, Sue Chang^9^, Andrew Blakely^10^, Danielle Deperalta^2^, Marc Reymond^11^, Amit Merchea^12^; Thanh Hue Dellinger^1^

^1^City of Hope National Medical Center, Department of Surgery; ^2^Northwell Health, Department of Surgery; ^3^City of Hope National Medical Center, Biostatistics; ^4^City of Hope National Medical Center, Medical Oncology; ^5^City of Hope Comprehensive Cancer Center, Beckman Research Institute; ^6^City of Hope National Medical Center, Department of Anesthesia; ^7^ Medical School Hanover, Institute of Pathology; ^8^Northwell Health Pathology; ^9^City of Hope Department of Pathology; ^10^National Institute of Health, U.S.A.; ^11^ University Hospital Tuebingen, Department of Surgery and Transplantation; ^12^Mayo Clinic, Surgery

**Background**: Prior studies have demonstrated the feasibility and safety of Pressurized intraperitoneal aerosolized chemotherapy (PIPAC) as a novel minimally invasive therapy for gastrointestinal and gynecologic peritoneal metastases. The goal of the present Phase 1 trial was to establish the safety and feasibility of PIPAC oxaliplatin in a highly chemotherapy refractory cohort of colorectal and appendiceal cancer patients.

**Methods**: Patients with peritoneal metastases from colorectal or appendiceal cancer underwent up to 3 PIPAC treatments using oxaliplatin (92 mg/m^2^) with a 6-week interval. Patients with bowel obstruction, extra-peritoneal metastases, or poor performance status (ECOG>2) were excluded. Apart from the first PIPAC cycle, the patients also received a sensitizing dose of 5FU/LV (400mg/m^2^) within 24 hours pre-PIPAC. Primary end point was safety as assessed by dose limiting toxicities (DLT) within 6 weeks of the first PIPAC. Secondary endpoints included safety with the addition of 5FU/LV, efficacy, surgical morbidity, technical failure rate, progression-free and overall survival, pharmacokinetics (PK), and quality of life assessment.

**Results**: A total of 12 patients were included: 8 colorectal; and 4 appendiceal. Median number of prior chemotherapy cycles was 2 (Interquartile range – IQR; 2-3). All patients were refractory to systemic oxaliplatin-based chemotherapy. Median time from diagnosis to PIPAC was 476 days (IQR; 309, 560) and Peritoneal Carcinomatosis Index was 28 (IQR; 19, 32). Six (55%) of eleven patients who completed protocol therapy completed 3 PIPAC cycles. No surgical complication or DLT was observed. Only two patients developed grade 3 treatment-related toxicity (abdominal pain and anemia). At the completion of PIPAC treatment 5 patients had stable disease and 5 had disease progression by imaging.

**Conclusions**: PIPAC with oxaliplatin is safe and feasible in a highly chemotherapy refractory cohort of

appendiceal and colorectal carcinomatosis patients with or without sensitizing 5-FU/ LV.

## ISSPP 2022 POSTER ABSTRACT 60.

### PHASE I DOSE-ESCALATION TRIAL DESIGN TO EVALUATE THE SAFETY AND TOLERABILITY OF DOCETAXEL PIPAC IN COMBINATION WITH FIRST-LINE SYSTEMIC THERAPY IN PATIENTS WITH GASTRIC CANCER PERITONEAL METASTASES

^1^Kevin Sullivan, ^2^Raghav Sundar, ^3^Chao, Joseph, ^4^Klempner, Samuel, ^3^Li, Daneng, ^5^Alexander Jung, ^6^Chang, Sue, ^6^Rifat Mannan, ^7^Paul Frankel, ^8^Wei Peng Yong, ^1^I. Benjamin Paz, ^1^Thanh Hue Dellinger, ^1^Mustafa Raoof, ^9^Jimmy Bok Yan So, ^1^Yuman Fong, ^1,10^Yanghee Woo

^1^City of Hope National Medical Center, Department of Surgery; ^2^National University of Singapore; ^3^City of Hope National Medical Center, Department of Medical Oncology; ^4^Massachusetts General Hospital, Medical Oncology; ^5^City of Hope National Medical Center, Department of Radiology; ^6^City of Hope National Medical Center, Department of Pathology; ^7^City of Hope National Medical Center, Biostatistics; ^8^National University Cancer Institute, ^10^City of Hope Comprehensive Cancer Center / Beckman Research Institute, U.S.A

**Introduction**: In gastric cancer (GC), failure of standard of care (SOC) first-line therapy often leads to progression of peritoneal metastases (PM) within 3 months. Pressurized intraperitoneal aerosolized chemotherapy (PIPAC) is a well-tolerated surgical drug delivery method with promising early phase trial results. However, the safety and efficacy of docetaxel PIPAC in GCPM is unknown. We propose to establish the maximum tolerated dose of docetaxel PIPAC added to SOC first-line therapy in GCPM.

**Methods**: GCPM patients who have received ≥ 3 months of first-line therapy consisting of intravenous oxaliplatin and fluorouracil (5-FU) plus leucovorin +/- trastuzumab or pembrolizumab are eligible. Patients are excluded if extraperitoneal disease, progression, contraindications for laparoscopy, poor performance status, or bowel obstruction are present. A cycle consists of SOC chemotherapy on days 1 and 14 with docetaxel PIPAC on day 28, followed by 14 days off. Cycles will be repeated with adverse event (AE) monitoring until progression, intolerance/toxicity, or 1 year. The dose escalation schedule follows the 3+3 design (lead-in cohort 50 mg/m^2^, then 75, 100, and 125 mg/m^2^) plus SOC chemotherapy. Expansion arm will allow pembrolizumab 200 mg or trastuzumab 8mg/kg then 6mg/kg per SOC.

**Results**: Primary endpoint are incidence and severity of AEs and dose limiting toxicity (DLT). Secondary endpoints include peritoneal tumor response (by peritoneal regression grade score, peritoneal carcinomatosis index score, imaging [RECIST 1.1]), progression free and overall survival rates, technical failure rate, and patient-reported outcomes. Longitudinal blood, urine and tissue specimens will be collected for translational correlatives including pharmacokinetics, circulating biomarkers, immune profiling, and single-cell transcriptomics.

**Conclusions:** This Phase 1 trial will establish the safety and tolerability of combination PIPAC and systemic therapy for GCPM in the first-line setting. Further, we expect to detect an early efficacy signal to inform phase II trial design.

## ISSPP 2022 POSTER ABSTRACT 61.

### SODIUM THIOSULFATE ABROGATES CISPLATIN-INDUCED NEPHROTOXICITY FROM HYPERTHERMIC INTRAPERITONEAL CHEMOTHERAPY (HIPEC) WITHOUT COMPROMISING SURVIVAL IN OVARIAN CANCER: RESULTS FROM A PROSPECTIVE CLINICAL TRIAL

^1^Nicole Lugo Santiago, ^1^Ernest Soyoung Han, ^1^Mustafa Raoof, ^2^Xiwei Wu, ^1^Hyejin Cho, ^1^Stephen Lee, ^1^Wei-Chien Lin, ^1^Jeff Feng-Hsu Lin, ^3^Daphne B. Stewart, ^4^Nora H. Ruel, ^1^Edward Wenge Wang, ^1^Isaac Benjamin Paz, ^1^Mark Tsuneo Wakabayashi, ^1^Lorna Rodriguez-Rodriguez, ^1^Mihaela C. Cristea, ^1^Thanh Hue Dellinger

^1^City of Hope National Medical Center, Department of Surgery, CA; ^2^City of Hope Beckman Research Institute, Duarte, CA; ^3^City of Hope National Medical Center, Department of Medical Oncology, CA; ^4^City of Hope National Medical Center, Biostatistics, CA

**Background**: Hyperthermic intraperitoneal chemotherapy (HIPEC) with cisplatin confers a survival benefit in epithelial ovarian cancer (EOC). Unfortunately, cisplatin is associated with significant renal toxicities. Sodium thiosulfate (ST) has been suggested as a nephroprotectant for patients undergoing HIPEC with cisplatin, though limited long-term survival data exist.

**Methods**: A feasibility trial (ClinicalTrials.gov: NCT01970722) evaluated safety outcomes of HIPEC with cisplatin 75 mg/m2 during optimal cytoreductive surgery (CRS) in patients with EOC and endometrial cancer (n = 40), with or without ST. Twenty-one patients received no sodium-thiosulfate (nST group), and nineteen patients received sodium thiosulfate (ST group). Toxicities were reported according to CTCAE v. 5. Progression-free (PFS) and overall survival (OS) was followed. Serum creatinine was measured preoperatively and postoperatively. Kaplan Meier and log-rank tests compared PFS and OS of patients between ST and nST groups. Normal tissue biopsies were collected intra-operatively immediately following HIPEC and cisplatin exposure in a subset of patients (n = 21), and profiled with transcriptomic sequencing to identify RNAseq signatures correlating with toxicities. Hierarchical cluster analyses identified distinct transcriptomic signatures in post-HIPEC normal samples of patients with renal AEs (rAEs) compared to no renal AEs (nrAEs).

**Results**: Forty patients had HIPEC at time of optimal CRS. Renal toxicities were higher in the nST group (no sodium thiosulfate) compared to the ST group. nST patients had 17% any grade, and 9% Grade 3 AEs for acute and chronic kidney injuries. In contrast, ST patients suffered 0% renal AEs. Mean postoperative serum Creatinine in the nST group was significantly higher than in the ST group: 1.5 vs. 0.6 (p-value-0.006). Median increase of serum creatinine from preoperative to postoperative value in the nST group was 53% compared to a 10% decrease in the ST group. Kaplan-Meier curves demonstrated similar PFS in primary ovarian cancer patients in the ST group (16.2 months, 6.4, 31.0) vs nST group (14.2 months, 5.6, NR) (p=NS). rAE patients demonstrated upregulation of immune signaling pathways (Toll-like receptor, Natural killer cell, Nod-like receptor); and downregulation of metabolic pathways. Top upregulated genes in rAE patients included immune (e.g. neutrophil) related genes, while downregulated genes included metabolism genes.

**Conclusions**: HIPEC-cisplatin induced renal toxicities are mitigated by the nephro-protectant sodium thiosulfate, without resultant differences in progression-free survival in ovarian cancer patients. Based on transcriptomic analysis of HIPEC treated normal tissues, the mechanisms of cisplatin-induced nephrotoxicity in HIPEC are immune-related and reflect reduced metabolism

## ISSPP 2022 POSTER ABSTRACT 62.

### TUMOR MICROENVIRONMENT CHANGES IN PERITONEAL TUMORS OF A PLATINUM-RESISTANT OVARIAN CANCER RESPONDER TO PRESSURIZED INTRAPERITONEAL AEROSOLIZED CHEMOTHERAPY (PIPAC): A SINGLE CELL TRANSCRIPTOMIC ANALYSIS

Thanh H. Dellinger^1^, Isaac Bishara^1^, Patrick A. Cosgrove^1^, Sumana Majumdar^1^, Adrian Kohut^1^, Raechelle Tinsley^2^, Melissa Eng^2^, Paul Frankel^1^, Sue Chang^1^, Aimin Li^1^, Wiebke Solass^2^, Mihaela C. Cristea^1^, Mustafa Raoof^1^, Andrea Bild^3^

^1^City of Hope National Medical Center, Duarte CA; ^2^City of Hope National Medical Center Institute of Pathology and Neuropathology University Hospital Tübingen, Eberhard-Karls-University Tübingen, Germany; ^2^ Institute of Pathology, University Bern, Switzerland; ^3^City of Hope Beckman Research Institute, CA

**Objectives**: Low grade serous (LGS) is a rare subtype in ovarian cancer (OC) with inherently platinum-resistant tumors. Patients with recurrent peritoneal metastases (PM) from LGS OC that are unresectable have poor prognoses. PIPAC is an intraperitoneal (IP) treatment that intensifies chemotherapy delivery to PM via drug nebulization and pressurization. PIPAC has demonstrated promising efficacy in platinum-resistant OC patients. We analyzed the molecular and tumor microenvironment (TME) changes of a PIPAC-treated LGS OC patient with platinum-resistant tumors.

**Methods**: A heavily pretreated, platinum-resistant recurrent LGS OC patient underwent PIPAC (aerosolized cisplatin 10.5 mg/m^2^ and doxorubicin 2.1 mg/m^2^, at 12 mmHg), via laparoscopy q6 weeks, for two cycles (NCT04329494). Tumor and normal peritoneum were biopsied immediately before and after each PIPAC. After cancer cell and nuclei isolation, sc-RNAseq was performed. 10X Genomics generated cDNA libraries were sequenced on Illumina HiSeq 2500 or NovaSeq 6000 instruments using 150 cycle paired-end sequencing at a depth of 10K reads/cell. Multiplex immunohistochemistry (IHC) was performed (quad staining PAX5-DAB/PD-L1/CD68/Tryptase; triple staining PD-1/CD8/CD3; double staining FOXP3-DAB/PD-1).

**Results**: The Peritoneal Carcinomatosis Index (PCI) reduced from 20 to 14 after one cycle, as demonstrated at Cycle #2. scRNAseq of post-PIPAC tumors demonstrated significantly upregulated immune and KRAS signaling pathways, compared to post-PIPAC normal tissues. Acute PIPAC-induced responses included upregulation of immune pathways (inflammatory response, complement, interferon-gamma response), hormonal signaling (androgen, estrogen late response), TNF- signaling via NF- B, apoptosis, and hypoxia pathways. PD-1 expression was increased in tumor infiltrating lymphocytes (TILs) within cancer islands.

**Conclusions**: PIPAC induces peritoneal tumor regression in LGS OC, possibly via modulation of TME, and upregulation of immune and KRAS signaling pathways; thus, suggesting potential future combination with MEK inhibitors and immunotherapies. le cell RNA sequencing.

